# Synthesis, Spectroscopy, Single-Crystal Structure Analysis and Antibacterial Activity of Two Novel Complexes of Silver(I) with Miconazole Drug

**DOI:** 10.3390/ijms22041510

**Published:** 2021-02-03

**Authors:** Karolina Stryjska, Izabela Korona-Glowniak, Lilianna Chęcińska, Joachim Kusz, Justyn Ochocki

**Affiliations:** 1 Department of Bioinorganic Chemistry, Chair of Medicinal Chemistry, Medical University of Lodz, Muszyńskiego 1, 90-151 Łódź, Poland; karolina.stryjska@umed.lodz.pl; 2 Department of Pharmaceutical Microbiology, Medical University of Lublin, Chodźki 1, 20-093 Lublin, Poland; iza.glowniak@umlub.pl; 3 Faculty of Chemistry, University of Lodz, Pomorska 163/165, 90-236 Łódź, Poland; lilianna.checinska@chemia.uni.lodz.pl; 4 Institute of Physics, University of Silesia, 75 Pułku Piechoty 1, 41-500 Chorzów, Poland; joachim.kusz@us.edu.pl

**Keywords:** synthesis, silver(I) complexes, NMR spectroscopy, IR spectroscopy, X-ray crystallography, antimicrobial activity

## Abstract

In a previous article, we reported on the higher toxicity of silver(I) complexes of miconazole [Ag(MCZ)_2_NO_3_ (**1**)] and [Ag(MCZ)_2_ClO_4_ (**2**)] in HepG2 tumor cells compared to the corresponding salts of silver, miconazole and cisplatin. Here, we present the synthesis of two silver(I) complexes of miconazole containing two new counter ions in the form of Ag(MCZ)_2_X (MCZ = 1-[2-(2,4-dichlorobenzyloxy)-2-(2,4-dichlorophenyl)ethyl]-1H-imidazole]; X = BF_4_^−^ (**3**), SbF_6_^−^ (**4**)). The novel silver(I) complexes were characterized by elemental analysis, ^1^H NMR, ^13^C NMR and infrared (IR) spectroscopy, electrospray ionization (ESI)-MS spectrometry and X-ray-crystallography. In the present study, the antimicrobial activity of all obtained silver(I) complexes of miconazole against six strains of Gram-positive bacteria, five strains of Gram-negative bacteria and yeasts was evaluated. The results were compared with those of a silver sulfadiazine drug, the corresponding silver salts and the free ligand. Silver(I) complexes exhibited significant activity against Gram-positive bacteria, which was much better than that of silver sulfadiazine and silver salts. The highest antimicrobial activity was observed for the complex containing the nitrate counter ion. All Ag(I) complexes of miconazole resulted in much better inhibition of yeast growth than silver sulfadiazine, silver salts and miconazole. Moreover, the synthesized silver(I) complexes showed good or moderate activity against Gram-negative bacteria compared to the free ligand.

## 1. Introduction

Currently, drug resistance to chemotherapeutics and antibiotics is a serious problem in modern medicine. Therefore, solving this problem has become a challenge for researchers. Despite growing public awareness and the development of treatment methods, prolonged antibiotic therapy and medicine abuse have contributed to an increase in the number of deaths caused by antibiotic-resistant bacterial strains [1]. This intensifies the need to search for new compounds with various mechanisms of action and potential antimicrobial properties. A literature review clearly shows the growing interest in the use of precious metals in medicine as an alternative in fighting infections. The latest research shows that new compounds containing elements such as gold, silver, nickel or ruthenium have promising potential and can be used as compounds with antimicrobial properties in the fight against infections [2,3]. Many researchers focus their attention on silver, which is used in medicine due to its strong antibacterial activity against Gram-positive and Gram-negative bacteria and fungi [4].

Silver and its compounds have been used in medicine since 4000 years B.C. [5,6]. The medical use of silver was documented in the literature as early as the 17th and 18th centuries [7]. Due to its properties, it was used to treat burns and skin ulcers, to treat stomach ulcers and to sterilize medical instruments, among other purposes. Silver and its compounds have also been used in the treatment of gonorrhea, tetanus and colds [8,9,10,11,12]. Silver nitrate solution was used during the Credé procedure to prevent gonorrhea conjunctivitis in newborns [13,14]. Silver proteinate, known as protargol and argyrol, has also been used in neonatal ophthalmology to treat neonatal conjunctivitis. Contrary to silver nitrate, silver proteinate was neither corrosive nor irritating [15,16,17]. Another substance used is the complex compound silver sulfadiazine, which is formed by combining two antibacterial substances: the sulfonamide group (sulfadiazine) and silver ion. Silver sulfadiazine is used in the form of a cream (Flamazine^®^) to combat fungal and bacterial infections and skin burns. However, its use is associated with the occurrence of side effects, such as allergic reactions, gastrointestinal reactions and slow wound healing [18,19,20,21,22].

Currently, many researchers are focusing on the synthesis of silver(I) complexes due to the low toxicity of silver ions in humans and the improved action of such compounds compared to popular antibiotics. In addition, coordination of different ligands to Ag(I) ions can achieve the prolonged release of Ag(I) ions at the site of infection to combat certain infections. Silver(I) complexes have been proven to exhibit antibacterial [23,24,25,26], antifungal [27,28,29,30] and anticancer [31,32,33,34,35,36,37] properties that are better than those of the free ligand.

**Figure 1:**
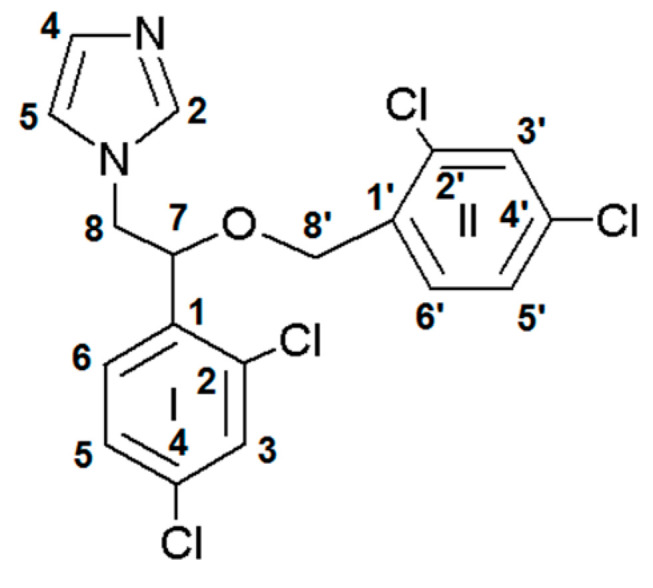
Chemical structure of miconazole.

Imidazole derivatives constitute an important class of compounds and have been studied for many years. Miconazole (Figure 1) was approved for use in 1971 and is included in the list of essential drugs of the World Health Organization [38,39]. It is a drug with a broad spectrum of antifungal activity. It is used externally in dermatophyte and fungal skin infections and vaginal candidiasis [40,41,42,43]. Miconazole is also bactericidal against various strains of Gram-positive aerobic bacteria. It is not active against Gram-negative bacteria [44,45].

The antibacterial mechanism of action of silver(I) metal complexes may be different, which is promising for slowing down the development of drug resistance in bacteria. Catalytic oxidation, reactions with the cell wall of bacteria, protein denaturation and binding to DNA, which leads to the death of bacteria, are indicated as methods of deactivation of bacteria by silver [46,47,48,49]. This provides a basis for continued research and the discovery of new compounds of silver(I) complexes.

In our previous article, we described the synthesis of two new silver(I) complexes of miconazole, [Ag(MCZ)_2_NO_3_ (**1**)] and [Ag(MCZ)_2_ClO_4_ (**2**)], and tested their cytotoxicity using Balb/c 3T3 and HepG2 cell lines [50]. The obtained results were promising and provided a broader horizon for silver(I) complexes.

Therefore, we synthesized new silver(I) complexes of miconazole containing new counter ions (BF_4_^−^ (**3**), SbF_6_^−^ (**4**)), which are described in this publication. In this paper, we present the synthesis of the newly obtained silver(I) complexes of miconazole. The obtained compounds were characterized by spectroscopic methods (^1^H NMR, ^13^C NMR, infrared (IR)), electrospray ionization (ESI)-MS, elemental analysis and X-ray measurements. In addition, we examined the antimicrobial activity of all silver(I) complexes of miconazole (**1**–**4**) that we developed so far against selected Gram-positive and Gram-negative bacteria and yeasts by calculating the minimum inhibitory concentration (MIC) and minimum bactericidal concentration (MBC) values.

## 2. Results and Discussion

### 2.1. Synthesis of [Ag(MCZ)_2_NO_3_] (***1***) and [Ag(MCZ)_2_ClO_4_] (***2***)

The synthesis of both complexes and their crystal structures were described in our previous article. Both silver(I) complexes are in monomeric forms. They consist of the appropriate counter ions (nitrate and perchlorate) and silver(I) ions coordinated by two miconazole ligands. Their spectroscopic properties (IR, ^1^H NMR, ESI-MS), X-ray diffraction analysis and cytotoxic potential against the HepG2 human liver cancer cell line were previously determined and reported [50].

**Scheme 1:**
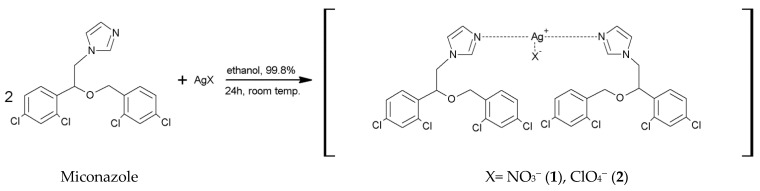
Synthesis of silver(I) complexes of miconazole (**1**) and (**2**).

Generally, both complexes were obtained via reaction of the corresponding silver salts (AgX = NO_3_^−^ (**1**), ClO_4_^−^ = (**2**)) with miconazole in a 1:2 molar metal-to-ligand ratio. A scheme for the synthesis of silver(I) complexes of miconazole is shown below (Scheme 1).

### 2.2. Synthesis of [Ag(MCZ)_2_BF_4_] (***3***) and [Ag(MCZ)_2_SbF_6_] (***4***)

**Scheme 2:**
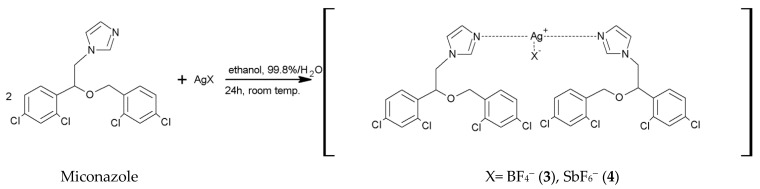
Synthesis of silver(I) complexes of miconazole (**3**) and (**4**).

The new silver(I) complexes of miconazole were synthesized in an easy process in a reaction of AgBF_4_ and AgSbF_6_ with miconazole (1:2) (Scheme 2) in water/ethanol. The complexes were obtained in good yield.

### 2.3. NMR Spectroscopy

The characteristics of the ^1^H NMR spectra for [Ag(MCZ)_2_NO_3_] (**1**) and [Ag(MCZ)_2_ClO_4_] (**2**) complexes were described in our previous work [50]. The ^1^H and ^13^C NMR spectra of the newly synthesized complexes and free miconazole ligand were recorded in CDCl_3_. Carbon and hydrogen atoms are numbered as shown in Figure 1.

In the spectrum of miconazole, signals appear at 7.26 and 7.27 ppm, which are attributed to H4 and H5 protons of the imidazole ring. The signals assigned to H3 and H3’ protons in the dichlorophenyl rings are observed in the spectrum of the free ligand at 7.38 and 7.48 ppm. The spectrum of the free ligand also shows signals at 7.32 and 7.33 ppm, which are assigned to H6’ and H5’ protons. For H6 and H5 protons, signals are recorded at 7.16 and 7.20. The aliphatic CH proton is recorded at 4.10 ppm, while for two aliphatic protons, CH_2_ signals between 4.27 and 4.52 ppm are observed. The signals recorded in the miconazole spectrum in this study are consistent with the literature values [51]. 

In the ^1^H NMR spectra of all silver(I) complexes of miconazole (**1**–**4**) that we obtained so far, slight shifts to a higher field are observed compared to the spectra of miconazole (Table 1). Moreover, in the silver(I) complex spectra, signals appear at 8.08 ppm for compound **1**, at 8.20 ppm for **2,** at 8.17 ppm for **3** and at 8.13 ppm for compound **4**. These signals can be assigned to the H2 proton in the imidazole ring. In the miconazole spectrum, this band is not recorded. A review of the literature shows that the slight shifts to the higher field for silver(I) complexes compared to the free ligand are not surprising. This indicates the effect of coordination of the ligand through the nitrogen atom of the imidazole ring with Ag(I) ions, only slightly reflected in the peripheral ring protons [52,53,54,55]. Spectra of the silver(I) complexes of miconazole **3** and **4** are presented in Supplementary Figure S1.

The ^13^C NMR spectroscopic data for **3** and **4** in comparison to the free ligand are presented in Table 2. The registered spectra of silver(I) complexes of miconazole and the free ligand are similar. In this case, slight shifts towards the higher field are also observed in relation to the ligand spectrum. The signals of the C2 carbons in the imidazole ring for compounds **3** and **4** appear at 140.40 and 140.41 ppm, respectively. In the spectrum of miconazole, this band appears at 138.31 ppm, which also confirms the connection of Ag(I) with the ligand through the nitrogen atom of the imidazole ring. ^13^C NMR spectra of **3**, **4** and miconazole are presented in Supplementary Figure S2.

### 2.4. IR Spectroscopy

The IR spectra of complexes **1** and **2** were characterized in our recent work [50]. In the miconazole spectrum, a band at 3151 cm^−1^ is observed, which is assigned to the CN vibration of the imidazole group [56]. This band for complexes **3** and **4** is located at 3180 (**4**) and 3182 cm^−1^ (**3**), respectively. In the free drug spectrum, a band at 3109 cm^−1^ corresponding to aromatic CH stretching [56] is observed, and in the complex spectra, these vibrations appear at 3129 (**3**) and 3137 cm^−1^ (**4**), respectively.

The spectrum of the free ligand has bands at 1589 and 1562 cm^−1^, which are attributed to C—C stretching vibrations of the dichlorobenzene groups. In the spectra of complexes **3** and **4**, these bands appear at 1590 cm^−1^ for **3** and **4** as well as 1561 (**4**) and 1562 cm^−1^ (**3**). The bands at 1505 cm^−1^ in the miconazole spectrum can be attributed to C—C stretching vibrations of the imidazole group and CH bending of the imidazole group, i.e., the C6 aliphatic part of the ligand [57]. The spectrum of miconazole also shows a band at 1468 cm^−1^, which is attributed to CH bending vibrations of two dichlorobenzene groups. In the spectra of silver(I) complexes with miconazole, this band is located at 1470 (**4**) and 1471 cm^−1^ (**3**). The band appearing in the miconazole spectrum at 1092 cm^−1^, attributable to the stretching vibrations of the C—O—C group [57], is found at 1094 (**3**) and 1095 cm^−1^ (**4**) in the spectrum of the complexes. The lack of significant band shifts originating from the C—O—C group for the silver(I) complexes indicates a lack of coordination by this group. In the spectra of silver(I) complexes, bands at 1728 cm^−1^ for compound **3** and 1729 cm^−1^ for **4** are observed. This band is not observed in the spectrum of the free ligand. It can be assumed that these bands are derived from the stretching vibrations of the C═N group of the imidazole nitrogen [50]. This indicates the connection of Ag(I) ions with miconazole by the nitrogen of the imidazole group. This is also confirmed by the X-ray structure of the obtained single crystals of silver(I) complexes **3** and **4**. Moreover, strong bands appear in the spectra of the complexes at 1071 cm^−1^ for compound **3** and 658 cm^−1^ for compound **4**, which is confirmed by the presence of BF_4_^−^ and SbF_6_^−^ anions [58]. In addition, a higher intensity of the bands is indicated in the spectra of the complexes compared to the spectrum of the free drug. The spectra of silver(I) complexes **3** and **4** are presented in Supplementary Figure S3.

### 2.5. Light Stability

The study was carried out for complexes of silver(I) with miconazole **1**–**4** and for the corresponding silver salts used for comparison purposes. The light stability of the complexes was monitored under light in an air atmosphere at room temperature to check their light stability under natural living conditions. Solutions of the complexes and silver salt at a concentration of 0.05 mol/L were applied to various substrates (paper, tissue paper and glass) and visually monitored for 0, 1, 4, 18, 24, 40, 48, 52, 60, 84 and 108 h.

AgNO_3_, AgClO_4_, AgBF_4_ and AgSbF_6_ slightly darkened after 1 h of exposure to light, while after 40 h, their complete blackening was observed on tissue paper and paper. On the glass, their decomposition was a bit slower. Silver(I) complexes of miconazole **1**–**4** remained unchanged on all substrates throughout the experiment. In summary, the stability of complex compounds **1**–**4** under light turned out to be much better than that of the corresponding silver salts. The silver salt decomposition proceeded rapidly, while the silver(I) complexes of miconazole remained unchanged (Supplementary Figure S4).

The results of the experiment carried out are promising for the possible use of compounds **1**–**4** as antibacterial agents for external application, e.g., in the form of ointments.

### 2.6. X-ray Single-Crystal Structure Determination

**Figure 2:**
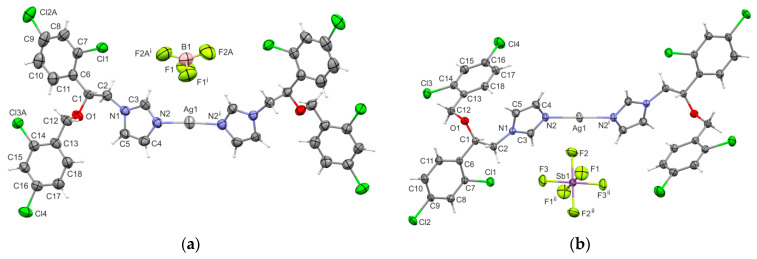
Molecular structures of Ag(I) complexes of miconazole with tetrafluoroborate (**3**) (**a**) and hexafluoroantimonate (**4**) (**b**) counter ions. The anisotropic displacement ellipsoids are drawn at the 30% probability level. For clarity, only the major components of the disordered atoms (F2A, Cl2A, Cl3A, H18) are shown. Symmetry codes: **3** (i) *−x*, *y*, *−z* + ½; **4** (i) *−x* + 1, *−y* + 2, *−z* + 1; (ii) *x* + 2, *−y* + 2, *−z* + 1.

The molecular structures of the new Ag(I) complexes of miconazole with two different counter ions, tetrafluoroborate (**3**) and hexafluoroantimonate (**4**), are displayed in Figure 2. 

In **3**, the Ag1 and B1 atoms are located on a two-fold symmetry axis, whereas in **4**, the Ag1 and Sb1 atoms lie on inversion centers. In both cases, the silver(I) ion is linearly coordinated by two symmetrically related miconazole ligands, which is confirmed by the N-Ag-N angle of 176.5(2)° for **3** and 180° for **4**. The Ag-N distances are 2.058(4)Å and 2.092(3) Å for **3** and **4**, respectively. The former distance is the shortest observed of the four complexes of silver(I) with miconazole ligands and different counter ions (**1**–**4**).

**Figure 3:**
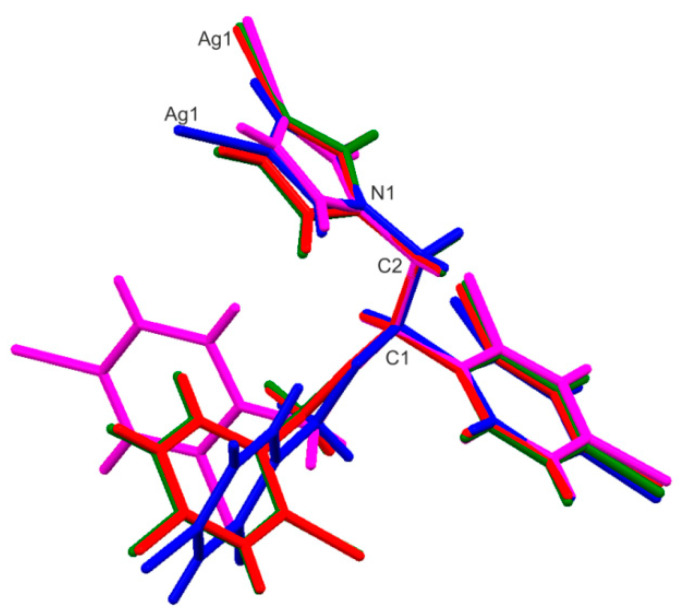
A superposition of miconazole ligands in Ag(I) complexes with different counter ions: nitrate (blue) (**1**), perchlorate (green) (**2**), tetrafluoroborate (red) (**3**), hexafluoroantimonate (magenta) (**4**). The best fit for C1, C2 and N1 atoms.

Figure 3 presents the overlay of four independent miconazole skeletons determined in silver(I) complexes **1**–**4**. As it is clearly seen, the molecular conformation of the miconazole ligands in complexes with perchlorate (**2**, green) and tetrafluoroborate (**3**, red) anions is identical. Noticeable differences are found in the orientations of the dichlorophenyl rings for miconazole moieties in the complexes with nitrate (**1**, blue) and hexafluoroantimonate (**4**, magenta) anions. Additionally, the orientation of the imidazole ring in complex **1** differs from the others.

Taking into account the configuration of the asymmetric atom C1, pairs of miconazole ligands within the cation molecule are designated as RR and SS for complexes **1–3**, which crystallize in the *C*2/c space group, whereas they are RS in the case of **4** due to the silver ion occupying the inversion center.

In complex **1**, the NO_3_^−^ anion binds strongly to the metal center, in contrast to the ClO_4_^−^, BF_4_^−^ and SbF_6_^−^ counter ions. In the new structures of **3** and **4**, there are no close Ag⋅⋅⋅F contacts.

**Figure 4:**
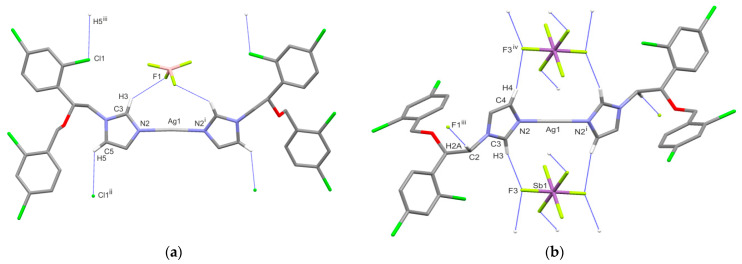
Hydrogen-bonding scheme in **3** (**a**) and **4** (**b**). In both cases, hydrogen atoms not involved in H-bonds are omitted for clarity. Symmetry codes: **3** (i) *−x, y, −z* + ½; (ii) *−x, y−*1, *z*; (iii) *−x, y* + 1, *z*; **4** (i) *−x* + 1*, −y* + 2*, −z* + 1*;* (iii) *−x* + 1, *−y* + 1, *−z* + 1; (iv) *x*−1, *y*, *z*.

**Figure 5:**
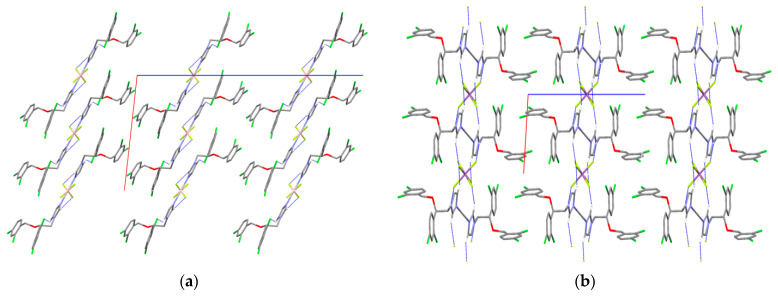
The crystal packing of **3** (**a**) and **4** (**b**) viewed along the crystallographic *b* axis.

Fluorine-containing anions do not participate in the coordination sphere of the Ag(I) ion, but both are involved in hydrogen bonds as acceptors. The geometrical details of the hydrogen bonds are summarized in Table 3. As shown in Figure 4a, the C3-H3-F1 hydrogen bond is observed between the cation and anion, whereas the C5-H5⋅⋅⋅Cl1 interaction links the cation molecules into a 1D-chain motif running parallel to the [10] direction. The crystal structure of complex **4** is dominated by three weak intermolecular C-H⋅⋅⋅F interactions (Figure 4b), which contribute to the formation of the molecular *ab*-layers. The packing diagrams are presented in Figure 5. Close inspection of the crystal packing reveals that in both analyzed structures, the aromatic π⋅⋅⋅π interactions can be found between the dichlorophenyl rings C13-C18 of the adjacent chain motifs in **3** and between neighboring layers in **4**. The centroid-to-centroid distances are 3.720(4)Å and 4.095(2)Å; the perpendicular distances from the centroid to the plane of the opposite ring are 3.472(2)Å and 3.665(2)Å for **3** and **4**, respectively.

### 2.7. Antimicrobial Activity

The antimicrobial activity of silver(I) complexes of miconazole, free ligand, silver sulfadiazine and the corresponding silver salts was tested against six strains of Gram-positive bacteria (*Staphylococcus aureus* ATCC 25923, *Staphylococcus epidermidis* ATCC 12228, *Micrococcus luteus* ATCC 10240, *Bacillus subtilis* ATCC 6633, *Bacillus cereus* ATCC 10876, *Enterococcus faecalis* ATCC 29212), five strains of Gram-negative bacteria (*Salmonella typhimurium* ATCC 14028, *Escherichia coli* ATCC 25922, *Proteus mirabilis* ATCC 12453, *Klebsiella pneumoniae* ATCC 13883, *Pseudomonas aeruginosa* ATCC 9027) and yeasts (*Candida glabrata* ATCC 90030, *Candida albicans* ATCC 102231, *Candida parapsilosis* ATCC 22019). Antimicrobial properties are expressed as MIC (minimum inhibitory concentration) and MBC/MFC (minimum bactericidal/fungicidal concentration) in μmol/L (Table 4). The antimicrobial activity of compounds **1**–**4** was compared with that of the antimicrobial and antifungal properties of the corresponding silver salts, miconazole and silver sulfadiazine.

All silver(I) complexes showed significantly higher activity against Gram-positive bacteria than that of silver sulfadiazine and silver salts. Their MIC activity was up to 23-fold higher than that of AgSD and the corresponding silver salts. They inhibited the growth of all Gram-positive bacteria at low concentrations. The most sensitive bacteria to the tested compounds **1**–**4** were staphylococci and micrococcus. It was shown that the synthesized compounds inhibited the growth of the *M. luteus* bacterial strain at concentrations of 0.49 μmol/L (for compound **1**) to 1.88 μmol/L (for compound **2**). The most active complex against this bacterium was complex **1**, which inhibited the growth of *M. luteus* at a 23-fold lower concentration than that of AgNO_3_ and 22-fold lower concentration than that of silver sulfadiazine. Other complexes showed 6- or 11-fold higher activity than that of silver sulfadiazine.

The tested complexes also revealed good antimicrobial activity against Gram-negative bacteria, but it was weaker than that against Gram-positive bacteria.

Out of the Gram-negative rods, the most sensitive was *P. aeruginosa*, the growth of which was inhibited most intensively by compounds **1**, **3** and **4** at concentrations of 31.24, 60.92 and 106.38 μmol/L, respectively. On the other hand, the activity of *E. coli* was best inhibited by compounds **1** and **2** at concentrations of 31.24 and 60.21 μmol/L. The MIC values of all complexes were slightly higher than those of the corresponding silver and sulfadiazine salts. The only exception was compound **1**, which showed better activity against *E. coli* than AgNO_3_ and silver sulfadiazine.

Moreover, the synthesized silver(I) complexes of miconazole were very active against yeasts. Even at low concentrations of 0.10–0.12 μmol/L, they inhibited the growth of *C. parapsilosis*, and at concentrations ranging from 0.83 to 1.95 μmol/L, they decreased the growth of *C. glabrata* and *C. albicans*. The corresponding silver salts and AgSD had significantly lower activity than that of complex compounds for all tested yeasts.

The MBC values for the tested complexes also confirmed that their activity against Gram-positive bacteria and yeasts was higher than that against Gram-negative bacteria in comparison to silver salts and silver sulfadiazine. The low values of the MBC/MIC ratio for complexes (**1**–**4**) suggested their bactericidal power against Gram-negative rods and Gram-positive bacilli in contrast to the higher values (8–16) for these complexes indicating bacteriostatic activities against staphylococci. A fungicidal effect (MFC/MIC ≤ 4) was found for Ag(MCZ)_2_NO_3_. The remaining complexes revealed fungistatic activity against the tested yeasts (MFC/MIC > 4), except for *C. albicans*.

The results for miconazole showed that its activity against Gram-positive bacteria was higher than that of the tested complexes. In contrast, the free ligand showed no inhibition of the growth of Gram-negative bacteria when it was below 1000 μmol/L, in contrast to all synthesized silver(I) complexes, which had moderate activity against these bacteria. Importantly, compared to the free drug, all of the complexes had 2-fold higher activity in relation to yeasts of *C. glabrata* and *C. albicans* and equal activity against *C. parapsilosis*. 

In summary, the tested complexes were more effective than salts and silver sulfadiazine against selected Gram-positive and yeast pathogens. In addition, the best antimicrobial properties were shown by complex **1**, which could potentially be used to treat serious skin infections, wounds and burns caused by the tested bacterial strains and yeasts as an alternative to the popularly used AgSD. Previous studies have indicated that antimicrobial activity increases with decreasing molecular weight of the compound [59]. Compound **1**, containing the NO_3_^−^ anion, has the lowest molecular weight among the silver(I) complexes presented in our work. It also seems interesting that the structure of this compound significantly differs from the other structures of Ag(I) complexes with miconazole, primarily in the connection of the nitrate ion with the silver(I) ion. In complex **1**, the NO_3_^−^ anion strongly binds to the metal center, in contrast to the counter ions BF_4_^−^, ClO_4_^−^ and SbF_6_^−^ counter ions. It has also been shown that silver(I) complexes with other drugs, e.g., naproxen, have strong antimicrobial properties and may be good candidates as antibacterial agents [60]. The studied silver(I) complexes of miconazole are much less active against Gram-negative bacteria than against Gram-positive bacteria. Similarly, previous studies have shown that the miconazole complexes of different metals are inactive against the Gram-negative bacteria *E. coli*, *P. aeruginosa* and *Yersinia pseudotuberculosis*, while they are effective against various strains of Gram-positive bacteria [59,61]. The different activity of silver(I) complexes against Gram-positive and Gram-negative bacteria may be caused by the difference in the structure of the cell walls of these bacteria and their interaction with the tested compounds (**1**–**4**). In the case of Gram-positive bacteria, their wall is made of peptidoglycan, while Gram-negative bacteria have an additional outer layer that contains a liposaccharide.

Previous studies have also indicated that different imidazole derivatives have good antimicrobial properties. The authors reported a good antibacterial effect of metronidazole silver(I) complexes against *S. epidermidis*, *S. aureus*, *P. aeruginosa* and *E. coli* [23]. The results of the research indicate that [Ag(MTZ)_2_NO_3_], [Ag(MTZ)_2_ClO_4_] and [Ag(MTZ)_2_BF_4_] compounds have significantly weaker activity in relation to *S. epidermidis* and *S. aureus* than that of the [Ag(MCZ)_2_NO_3_], [Ag(MCZ)_2_ClO_4_] and [Ag(MCZ)_2_BF_4_] compounds described in our article. *S. aureus* growth inhibition by silver(I) complexes of miconazole was 25-fold higher than that by silver(I) complexes of metronidazole. On the other hand, the antibacterial activity of compounds **1**–**3** against S*. epidermidis* was 10-fold better than that of compounds with metronidazole. The above comparison shows that the appropriate choice of the free ligand may lead to better antimicrobial properties.

## 3. Experimental Section

### 3.1. Chemicals and Reagents

Analytical standards of silver tetrafluoroborate (CAS:14104-20-2; molecular weight: 194 g/mol), silver hexafluoroantimonate (CAS: 26042-64-8; molecular weight: 343 g/mol) and silver sulfadiazine (CAS: 22199-08-2; molecular weight: 357 g/mol) were purchased from Sigma Aldrich (Poznań, Poland). Miconazole (CAS: 22946-47-8; molecular weight 416 g/mol) was purchased from Alfa Aesar (Kandel, Germany). All other chemicals were purchased from commercial suppliers and were of the highest available purity.

### 3.2. Synthesis 

#### 3.2.1. Synthesis of [Ag(Miconazole)_2_BF_4_] (**3**)

AgBF_4_ (0.5 mmol, 0.100 g) was dissolved in 5 mL of distilled water and was added to a miconazole (MCZ) ethanolic solution (miconazole (1 mmol, 0.416 g) in 10 mL of ethanol). The mixture was stirred for 24 h at room temperature. The precipitate was filtered off and washed in portions of diethyl ether. Crystals suitable for X-ray diffraction were obtained by diffusion in a tube with an aqueous solution of AgBF_4_ (0.1 mmol, 3 mL) at the bottom, methanol (2 mL) in the middle and a methanol solution of miconazole (0.2 mmol, 2 mL) on the top. M.w. 1026.87 g/mol; yield: (0.42 g, 82%); M.p. 161–164 °C. ESI-MS (methanol) m/z: 938.87 [Ag(MCZ)_2_]^+^. El. anal. Measured (calc.%): C—42.17 (42.11); H—2.46 (2.74); N—5.47 (5.47). ^1^H NMR (600 MHz, CDCl_3_) δ (ppm): 4.18 (m, 4H, 2 × N—CH_2_), 4.20 (m, 2H, 2 × CH—O), 4.56 (s, 4H, 2 × CH_2_—O), 7.07 (s, 2H, 2 × CH═C), 7.29 (s, 2H, 2 × CH═C), 7.33 (m, 2H, 2 × CH—N), 7.34 (m, 2H, 2 × CH—N), 7.35 (m, 2H, 2 × CH═C), 7.39 (s, 2H, 2 × CH═C), 7.41 (s, 2H, 2 × CH═C), 7.49 (d, 2H, 2 × CH═C, J = 1.90 Hz), 8.17 (s, 2H, 2 × CH—N). ^13^C NMR (600 MHz, CDCl_3_) δ (ppm): 140.40 (N═C—N), 134.61 (C—CH), 134.40 (C—Cl), 134.31 (C—Cl), 133.72 (C═CH), 133.67 (C—Cl), 131.38 (C—Cl), 129.82 (CH═C), 129.56 (CH—CH), 129.56 (CH═C), 129.30 (CH═C), 129.14 (CH—CH), 128.42 (CH—CH), 127.89 (CH═CH), 121.50 (CH—N), 76.95 (CH—O), 67.85 (CH_2_—O), 50.97 (CH_2_—N). IR (KBr) ν_max_/cm^−1^: 3129, 3094, 2975 (C—H) 1728 (C═N), 1590, 1562 (C—C, C═C), 1526 (C—C, C—H, C═C), 1471 (C—H, C═C), 1438 (C═C), 1248, 1139, 1120, 1094 (C—O—C), 1071 (BF_4_), 849, 815 (C—Cl). 

#### 3.2.2. Synthesis of [Ag(Miconazole)_2_SbF_6_] (**4**)

To an ethanolic solution of miconazole (1 mmol, 0.416 g, in 10 mL of ethanol) was added dropwise an aqueous AgSbF_6_ solution (0.5 mmol, 0.172 g in 5 mL in water). The mixture was stirred on a magnetic stirrer for 24 h at room temperature. The product was filtered off and washed in diethyl ether. Colorless crystals suitable for X-ray diffraction were grown in pure acetonitrile at room temperature. M.w. 1175.77 g/mol; yield: (0.38 g, 65%); M.p. 157–161 °C. ESI-MS (methanol) *m/z*: 938.87 [Ag(MCZ)_2_]^+^. El. anal. Measured (calc.%): C—36.76 (36.78); H—2.11 (2.39); N—4.76 (4.77). ^1^H NMR (600 MHz, CDCl_3_) δ (ppm): 4.17 (m, 4H, 2 × N—CH_2_), 4.20 (m, 2H, 2 × CH—O), 4.56 (s, 4H, 2 × CH_2_—O), 7.05 (s, 2H, 2 × CH═C), 7.29 (s, 2H, 2 × CH═C), 7.33 (m, 2H, 2 × CH—N), 7.34 (m, 2H, 2 × CH—N), 7.36 (m, 2H, 2 × CH═C), 7.41 (s, 2H, 2 × CH═C), 7.42 (s, 2H, 2 × CH═C), 7.49 (d, 2H, 2 × CH═C, J = 1.90 Hz), 8.13 (s, 2H, 2 × CH—N). ^13^C NMR (600 MHz, CDCl_3_) δ (ppm): 140.41 (N═C—N), 134.61 (C—CH), 134.39 (C—Cl), 134.31 (C—Cl), 133.72 (C═CH), 133.67 (C—Cl), 131.38 (C—Cl), 129.81 (CH═C), 129.57 (CH—CH), 129.57 (CH═C), 129.30 (CH═C), 129.15 (CH—CH), 128.42 (CH—CH), 127.89 (CH═CH), 121.49 (CH—N), 76.95 (CH—O), 67.85 (CH_2_—O), 50.97 (CH_2_—N). IR (KBr) ν_max/_cm^−1^:3180, 3137, 3061, 2957 (C—H) 1729 (C═N), 1590, 1562 (C—C, C═C), 1526 (C—C, C—H, C═C), 1470 (C—H, C═C), 1437 (C═C), 1248, 1139, 1119, 1095 (C—O—C), 848, 819 (C—Cl), 658 (SbF_6_).

### 3.3. Light Stability

The light stability of the compounds (**1**–**4**) was studied in normal light at room temperature. Silver(I) complexes of miconazole and their corresponding silver salts were dissolved in water to a volume of 2.5 µmol (50 µL) and then applied to impregnate various substrates: tissue paper, paper and glass. The concentration of each solution was 0.05 M. 

The stability of all compounds was monitored visually for 108 h, and photographs were acquired after 0, 1, 4, 18, 24, 40, 48, 52, 60, 84 and 108 h. The photos are shown in Supplementary Figure S4.

### 3.4. X-ray Single-Crystal Diffraction

The single-crystal X-ray diffraction experiments for silver(I) complexes **3** and **4** were carried out using a SuperNova diffractometer equipped with a microfocus X-ray tube, optimized multi-layer optics for MoKα (*λ* = 0.71073Å) radiation and an Atlas CCD detector. Measurements were carried out at room temperature. Control of the measurement procedure and data reduction were performed by CrysAlis^Pro^ software (version 1.171.38.41q; Rigaku Oxford Diffraction, 2015); the same program was used to determine and refine the lattice parameters. For integration of the collected data and correction of Lorentzian and polarization effects, the CrysAlis RED program was used (version 1.171.38.41q; Rigaku Oxford Diffraction, 2015) [62]. The structures were solved with SHELXT [63] and refined with SHELXL-2018/3 [64]. Hydrogen atoms were placed in idealized positions and refined as riding atoms. Experimental details, as well as a summary of the crystallographic data and structure refinement, are given in Table 5. 

In structure 3, the Cl2 and F2 atoms were found to be disordered. The final occupation factors kA:kB were refined to 0.60(5): 0.40(5) for the Cl2 atom and 0.553(13): 0.447(13) for the F2 atom, respectively. The ellipsoids of the A and B components were modeled. Bond-length restraints were applied to all C-Cl (C9-Cl2A, C9-Cl2B) and B-F (B1-F1, B1-F2A, B1-F2B) bonds involving the disordered atoms.

Additionally, a high residual-density peak around atom H18 was revealed during the refinement of **3**. Given the disorder of the chlorine Cl3 atom over two related *ortho* positions in the phenyl ring, two sets of split sites were used for the following atoms: Cl3B/Cl3A and H14/H18. The site-occupation factors are 0.845(5) for Cl3A/H18 and 0.155(5) for Cl3B/H14. The distances between atom pairs C14-Cl3A and C18-Cl3B were restrained to be equal. 

The graphical material for structural studies was generated with the Mercury program [65]. Supplementary crystallographic data for this paper can be found at the Cambridge Crystallographic Data Center under the depository numbers CCDC 2054546 (**3**) and 2054547 (**4**).

### 3.5. Chemistry

The melting points of all silver(I) complexes of miconazole were determined with a Böetius apparatus. Elemental analysis (C, H, N) was performed using the Perkin Elmer 2400 Series II Analyzer (PerkinElmer, Waltham, MA, USA). Electrospray ionization mass spectra (ESI-MS) were registered on a Varian 500-MS LC Trap (Varian Inc., Palo Alto, CA, USA). Infrared spectra were recorded in the range 4000–400 cm^−1^ on a Bruker Alpha-T spectrophotometer (Bruker Corporation, Billerica, MA, USA) using KBr pellets. ^1^H and ^13^C NMR spectra were collected with a Bruker advance III 600 MHz spectrophotometer (Bruker Corporation, Billerica, MA, USA) using CDCl_3_ as a solvent.

### 3.6. Antibacterial Activity Studies

All tested compounds were screened for antibacterial and antifungal activities using a micro-dilution broth method according to the European Committee on Antimicrobial Susceptibility Testing—EUCAST (www.eucast.org) using Mueller–Hinton broth and RPMI 1640 (Roswell Park Memorial Institute Medium) supplemented with 2% glucose and MOPS (3-(*N*-morpholino) propanesulfonic acid) for the growth of bacteria and fungi, respectively. The minimal inhibitory concentration (MIC) and minimal bactericidal/fungicidal concentration (MBC/MFC) of the tested complexes were evaluated for the panel of the reference microorganisms from American Type Culture Collection (ATCC), including Gram-negative bacteria (*Escherichia coli* ATCC 25922, *Salmonella* Typhimurium ATCC14028, *Klebsiella pneumoniae* ATCC 13883, *Pseudomonas aeruginosa* ATCC 9027, *Proteus mirabilis* ATCC 12453), Gram-positive bacteria (*Staphylococcus aureus* ATCC 25923, *Staphylococcus epidermidis* ATCC 12228, *Micrococcus luteus* ATCC 10240, *Enterococcus faecalis* ATCC 29212, *Bacillus subtilis* ATCC 6633, *Bacillus cereus* ATCC 10876) and fungi (*Candida albicans* ATCC 10231, *Candida parapsilosis* ATCC 22019, *Candida glabrata* ATCC 90030). The antimicrobial assays were performed in the same manner as in our previous research [66]. Each experiment was repeated in triplicate. Representative data are presented.

## 4. Conclusions

In this article, we present the continuation of biological research on the previously obtained complex compounds Ag(MCZ)_2_NO_3_ and Ag(MCZ)_2_ClO_4_, as well as propose the synthesis of two new silver complex compounds containing tetrafluoroborate and hexafluoroantimonate counter ions with the biologically active ligand miconazole. Their method of synthesis is simple and reproducible, which, combined with the promising results, shows great potential for their practical application in the treatment of bacterial and fungal infections. Their antibacterial and antifungal activities against six Gram-positive bacteria, five Gram-negative strains and fungi were assessed in comparison with the drug silver sulfadiazine, the ligand and the corresponding silver salts.

In our work, all synthesized silver(I) complexes (**1**–**4**) show much better activity in inhibiting the growth of Gram-positive bacteria and yeasts in comparison to silver sulfadiazine and silver salts. Moreover, against Gram-negative bacteria, their activity is better than that of miconazole. Furthermore, we can conclude that our compounds may be a better alternative to miconazole against yeast infections, including *C. albicans*.

Our study also shows that the presence of certain counter ions influences antimicrobial activity. The Ag(I) complex containing the nitrate counter ion is best at inhibiting the growth of Gram-positive and Gram-negative bacteria and yeasts.

Considering the problem of increasing drug resistance to popular antibiotics and drugs, all of our synthesized silver(I) complexes have great potential and can be good candidates for combating infections caused by bacteria and fungi.

## Figures and Tables

**Table 1:** Proton chemical shifts in ppm for miconazole and complex compounds at room temperature in CDCl_3_.

	Miconazole	Ag(MCZ)_2_NO_3_ [50]	Ag(MCZ)_2_ClO_4_ [50]	Ag(MCZ)_2_BF_4_	Ag(MCZ)_2_SbF_6_
Proton	Chemical Shift (ppm)	Chemical Shift (ppm)	Chemical Shift (ppm)	Chemical Shift (ppm)	Chemical Shift (ppm)
Imidazole					
H2	-	8.08	8.20	8.17	8.13
H4	7.26	7.32	7.33	7.33	7.33
H5	7.27	7.34	7.34	7.34	7.34
2,4-Dichlorophenyl					
(Ring I) H3	7.37	7.40	7.40	7.41	7.42
(Ring I) H5	7.20	7.29	7.29	7.29	7.29
(Ring I) H6	7.16	7.08	7.09	7.07	7.05
(Ring II) H3′	7.49	7.49	7.49	7.49	7.49
(Ring II) H5′	7.33	7.37	7.39	7.39	7.41
(Ring II) H6′	7.32	7.36	7.35	7.35	7.36
Aliphatic-CH	4.10	4.16	4.19	4.20	4.20
2-Aliphatic-CH_2_	4.27–4.52	4.29–4.55	4.32–4.56	4.33–4.56	4.34–4.56

**Table 2:** ^13^C NMR spectroscopy data for silver(I) complexes and the free ligand.

	Miconazole	Ag(MCZ)_2_BF_4_	Ag(MCZ)_2_SbF_6_
Carbon	Chemical Shift (ppm)	Chemical Shift (ppm)	Chemical Shift (ppm)
Imidazole			
C2	138.31	140.40	140.41
C4	120.43	121.50	121.49
C5	125.76	127.89	127.89
(Ring I) C1	135.01	134.61	134.61
(Ring I) C2	134.55	134.40	134.39
(Ring I) C3	129.74	129.82	129.81
(Ring I) C4	134.09	134.31	134.31
(Ring I) C5	129.07	129.56	129.57
(Ring I) C6	128.31	129.14	129.15
(Ring II) C1′	133.58	133.72	133.72
(Ring II) C2′	133.53	133.67	133.67
(Ring II) C3′	129.35	129.56	129.57
(Ring II) C4′	134.13	131.38	131.38
(Ring II) C5′	128.65	129.30	129.30
(Ring II) C6′	127.86	128.42	128.42
C7	77.49	76.95	76.95
C8	50.42	50.97	50.97
C8′	67.79	67.85	67.85

**Table 3:** Hydrogen bonding geometry (Å, °) for silver(I) complexes **3** and **4**.

H-Bond	D–H	H⋅⋅⋅A	D⋅⋅⋅A	D–H⋅⋅⋅A
**3**				
C3–H3⋅⋅⋅F1	0.93	2.53	3.292(7)	140
C5–H5⋅⋅⋅Cl1^ii^	0.93	2.80	3.434(5)	126
**4**				
C2–H2A⋅⋅⋅F1^iii^	0.97	2.37	3.313(4)	163
C3–H3⋅⋅⋅F1	0.93	2.35	3.221(4)	156
C4–H4⋅⋅⋅F3^iv^	0.93	2.61	3.478(4)	156

Symmetry codes: **3** (ii) −*x*, *y*−1, *z*; **4** (iii) *−x* + 1, *−y* + 1, *−z* + 1; (iv) *x*−1, *y*, *z*.

**Table 4:** Antimicrobial activity defined as minimal inhibitory concentration (MIC) and minimal bactericidal/fungicidal concentration (MBC/MFC) of silver(I) complexes, silver salts and reference compounds—miconazole (MCZ) and silver-sulfadiazine (AgSD).

Microorganism	Ag(MCZ)_2_NO_3_		AgNO_3_		Ag(MCZ)_2_ClO_4_		AgClO_4_		Ag(MCZ)_2_BF_4_		AgBF_4_	
	(μmol/L)		(μmol/L)		(μmol/L)		(μmol/L)		(μmol/L)		(μmol/L)	
**Gram-positive bacteria**	**MIC**	**MBC**	**MIC**	**MBC**	**MIC**	**MBC**	**MIC**	**MBC**	**MIC**	**MBC**	**MIC**	**MBC**
*S. aureus* ATCC 25923	1.95	15.57	22.81	45.61	3.76	30.15	18.84	37.68	1.90	30.51	20.10	80.41
*S. epidermidis* ATCC 12228	1.95	15.57	5.73	11.40	3.76	30.15	9.42	75.36	1.90	30.51	5.05	40.21
*M. luteus* ATCC 10240	0.49	15.57	11.40	22.81	1.88	7.51	18.84	75.36	0.96	7.60	10.05	80.41
*B. subtilis* ATCC 6633	3.89	7.78	22.81	45.61	7.51	15.03	18.84	75.36	7.60	30.51	20.10	40.21
*B. cereus* ATCC 10876	7.78	15.57	91.23	91.23	15.03	60.21	37.68	151.21	15.20	30.51	80.41	80.41
*E. faecalis* ATCC 29212	15.57	31.24	45.61	91.23	30.15	120.42	37.68	151.21	30.51	60.92	40.21	80.41
**Gram-negative bacteria**	**MIC**	**MBC**	**MIC**	**MBC**	**MIC**	**MBC**	**MIC**	**MBC**	**MIC**	**MBC**	**MIC**	**MBC**
*S.* Typhimurium ATCC 14028	62.38	62.38	22.81	91.23	120.42	120.42	18.84	37.68	121.83	243.66	20.10	40.21
*E. coli* ATCC 25922	31.24	31.24	45.61	45.61	60.21	60.21	75.36	75.36	121.83	121.83	80.41	80.41
*P. mirabilis* ATCC 12453	62.38	62.38	91.23	91.23	60.21	60.21	151.21	151.21	121.83	121.83	80.41	80.41
*K. pneumoniae* ATCC 13883	62.38	62.38	45.61	45.61	120.42	120.42	37.68	75.36	121.83	121.83	40.21	40.21
*P. aeruginosa* ATCC 9027	31.24	62.38	45.61	45.61	120.42	120.42	37.68	151.21	60.92	121.83	40.21	40.21
**Yeasts**	**MIC**	**MFC**	**MIC**	**MFC**	**MIC**	**MFC**	**MIC**	**MFC**	**MIC**	**MFC**	**MIC**	**MFC**
*C. glabrata* ATCC 90030	1.95	7.78	11.40	22.81	1.88	30.15	9.42	37.68	1.90	15.20	10.05	20.10
*C. albicans* ATCC 102231	0.98	1.95	11.40	91.23	1.88	3.76	9.42	75.36	1.90	3.80	20.10	80.41
*C. parapsilosis* ATCC 22019	0.12	0.49	11.40	91.23	0.12	1.88	4.73	75.36	0.12	1.90	10.05	80.41
**Microorganism**	**Ag(MCZ)_2_SbF_6_**		**AgSbF_6_**		**MCZ**		**AgSD**					
	**(** **μmol/L** **)**		**(** **μmol/L** **)**		**(** **μmol/L** **)**		**(** **μmol/L** **)**					
**Gram-positive bacteria**	**MIC**	**MBC**	**MIC**	**MBC**	**MIC**	**MBC**	**MIC**	**MBC**				
*S. aureus* ATCC 25923	3.32	26.64	22.74	45.48	1.18	9.38	21.85	43.70				
*S. epidermidis* ATCC 12228	3.32	26.64	5.69	22.74	1.18	9.38	43.70	43.70				
*M. luteus* ATCC 10240	1.66	6.64	11.37	45.48	1.18	1.18	10.92	87.68				
*B. subtilis* ATCC 6633	13.28	26.64	22.74	22.74	4.69	4.69	10.92	21.85				
*B. cereus* ATCC 10876	13.28	53.19	91.25	182.22	4.69	4.69	43.70	350.14				
*E. faecalis* ATCC 29212	26.64	106.38	45.48	91.25	9.38	18.75	87.68	87.68				
**Gram-negative bacteria**	**MIC**	**MBC**	**MIC**	**MBC**	**MIC**	**MBC**	**MIC**	**MBC**				
*S.* Typhimurium ATCC 14028	212.77	212.77	22.74	45.48	>1000	Nd	43.70	87.68				
*E. coli* ATCC 25922	212.77	212.77	91.25	91.25	>1000	Nd	43.70	87.68				
*P. mirabilis* ATCC 12453	212.77	212.77	91.25	91.25	>1000	Nd	21.85	43.70				
*K. pneumoniae* ATCC 13883	212.77	212.77	45.48	45.48	>1000	Nd	43.70	87.68				
*P. aeruginosa* ATCC 9027	106.38	106.38	45.48	45.48	>1000	Nd	21.85	43.70				
**Yeasts**	**MIC**	**MFC**	**MIC**	**MFC**	**MIC**	**MFC**	**MIC**	**MFC**				
*C. glabrata* ATCC 90030	0.83	13.28	11.37	22.74	4.69	18.75	10.92	43.70				
*C. albicans* ATCC 102231	1.66	3.32	11.37	91.25	4.69	9.38	10.92	43.70				
*C. parapsilosis* ATCC 22019	0.10	0.83	11.37	91.25	0.07	1.18	5.46	43.70				

**Table 5:** Crystallographic data for **3** and **4**.

	3	4
Empirical formula	C_36_H_28_AgCl_8_N_4_O_2_·BF_4_	C_36_H_28_AgCl_8_N_4_O_2_·SbF_6_
Formula weight	1026.90	1175.84
Crystal system	Monoclinic	Triclinic
Space group	*C*2/c	*P* $\bar{1}$
*a* (Å)	15.6809(10)	8.3845(6)
*b* (Å)	8.5678(5)	8.8813(5)
*c* (Å)	30.601(2)	14.9005(11)
α (°)	90.000	91.261(5)
β (°)	96.353(5)	93.497(5)
γ (°)	90.000	110.570(5)
*V* (Å^3^)	4086.0(5)	1035.77(12)
*Z*	4	1
*T* (K)	293(1)	293(1)
*F* (000)	2048	576
*D_x_* (g∙cm^−3^)	1.669	1.885
*µ* (mm^−1^)	1.074	1.71
Wavelength (Å)	0.71073	0.71073
*θ* range (°)	3.1–27.0	3.2–30.0
Measured reflections	17115	11524
Unique reflections	4455	6013
Observed reflections [*I* > 2*σ* (*I*)]	2961	4135
Completeness to *θ*_max_ (%)	99.8	99.4
Parameters/restraints	284/47	265/0
*R* [*F*^2^ > 2*σ* (*F*^2^)]	0.065	0.040
*wR* (all data)	0.196	0.101
*S*	1.06	1.01

## Data Availability

The data that support the findings of this study are available from the corresponding author upon reasonable request.

## Supplementary Materials

Supplementary Materials can be found at https://www.mdpi.com/1422-0067/22/4/1510/s1.
