# Supplementary material for: Synthesis, Spectroscopy, Single-Crystal Structure Analysis and Antibacterial Activity of Two Novel Complexes of Silver(I) with Miconazole Drug
Source: Int J Mol Sci. 2021 Feb 3;22(4):1510. doi: 10.3390/ijms22041510 (PMC7919260; doi:10.3390/ijms22041510)

checkCIF/PLATON report

Structure factors have been supplied for datablock(s) complex3, complex4

THIS REPORT IS FOR GUIDANCE ONLY. IF USED AS PART OF A REVIEW PROCEDURE FOR PUBLICATION, IT SHOULD NOT REPLACE THE EXPERTISE OF AN EXPERIENCED CRYSTALLOGRAPHIC REFEREE.

No syntax errors found. CIF dictionary Interpreting this report

Datablock: complex3

```
Bond precision:  C-C = 0.0090 A          Wavelength=0.71073

Cell:            a=15.6809(10)     b=8.5678(5)        c=30.601(2)
                 alpha=90          beta=96.353(5)     gamma=90
Temperature:     293 K
```

	Calculated	Reported
Volume	4086.0(4)	4086.0(5)
Space group	C 2/c	C 2/c
Hall group	-C 2yc	-C 2yc
Moiety formula	C36 H28 Ag Cl8 N4 O2, B F4	C36 H28 Ag Cl8 N4 O2, B F4
Sum formula	C36 H28 Ag B Cl8 F4 N4 O2	C36 H28 Ag B Cl8 F4 N4 O2
Mr	1026.90	1026.90
Dx,g cm-3	1.669	1.669
Z	4	4
Mu (mm-1)	1.074	1.074
F000	2048.0	2048.0
F000’	2049.91	
h,k,lmax	20,10,39	20,10,39
Nref	4463	4455
Tmin,Tmax	0.798,0.968	0.374,1.000
Tmin’	0.798	

```
Correction method= # Reported T Limits: Tmin=0.374 Tmax=1.000
AbsCorr = MULTI-SCAN

Data completeness= 0.998          Theta(max)= 26.999

R(reflections)= 0.0653( 2961)     wR2(reflections)= 0.1956( 4455)

S = 1.061                  Npar= 284
```

The following ALERTS were generated. Each ALERT has the format
test-name_ALERT_alert-type_alert-level.
Click on the hyperlinks for more details of the test.

Alert level C

```
PLAT234_ALERT_4_C Large Hirshfeld Difference Cl3B     --C18      .       0.23 Ang.
PLAT234_ALERT_4_C Large Hirshfeld Difference F2B      --B1       .       0.21 Ang.
PLAT242_ALERT_2_C Low    'MainMol' Ueq as Compared to Neighbors of        C18 Check
PLAT244_ALERT_4_C Low    'Solvent' Ueq as Compared to Neighbors of         B1 Check
PLAT260_ALERT_2_C Large Average Ueq of Residue Including        F1      0.137 Check
PLAT334_ALERT_2_C Small Aver. Benzene C-C Dist C6       -C11             1.37 Ang.
PLAT334_ALERT_2_C Small Aver. Benzene C-C Dist C13      -C18             1.36 Ang.
PLAT342_ALERT_3_C Low Bond Precision on  C-C Bonds ...............      0.009 Ang.
PLAT906_ALERT_3_C Large K Value in the Analysis of Variance ......      7.728 Check
PLAT910_ALERT_3_C Missing # of FCF Reflection(s) Below Theta(Min).          8 Note
```

Alert level G

```
PLAT002_ALERT_2_G Number of Distance or Angle Restraints on AtSite         11 Note
PLAT003_ALERT_2_G Number of Uiso or Uij Restrained non-H Atoms ...          5 Report
PLAT083_ALERT_2_G SHELXL Second Parameter in WGHT  Unusually Large      11.33 Why ?
PLAT176_ALERT_4_G The CIF-Embedded .res File Contains SADI Records          3 Report
PLAT177_ALERT_4_G The CIF-Embedded .res File Contains DELU Records          3 Report
PLAT178_ALERT_4_G The CIF-Embedded .res File Contains SIMU Records          3 Report
PLAT186_ALERT_4_G The CIF-Embedded .res File Contains ISOR Records          3 Report
PLAT199_ALERT_1_G Reported _cell_measurement_temperature ..... (K)        293 Check
PLAT200_ALERT_1_G Reported   _diffrn_ambient_temperature ..... (K)        293 Check
PLAT301_ALERT_3_G Main Residue  Disorder ..............(Resd  1  )         8% Note
PLAT302_ALERT_4_G Anion/Solvent/Minor-Residue Disorder (Resd  2  )        40% Note
PLAT432_ALERT_2_G Short Inter X...Y Contact  Cl3B     ..C10              3.05 Ang.
                                            -1/2+x,-1/2+y,z  =      3_445 Check
PLAT793_ALERT_4_G Model has Chirality at C1          (Centro SPGR)          R Verify
PLAT860_ALERT_3_G Number of Least-Squares Restraints .............         47 Note
PLAT941_ALERT_3_G Average HKL Measurement Multiplicity ...........        3.8 Low
PLAT955_ALERT_1_G Reported (CIF) and Actual (FCF) Lmax Differ by .          1 Units
PLAT965_ALERT_2_G The SHELXL WEIGHT Optimisation has not Converged     Please Check
PLAT978_ALERT_2_G Number C-C Bonds with Positive Residual Density.          0 Info
```

```
   0 ALERT level A = Most likely a serious problem - resolve or explain
   0 ALERT level B = A potentially serious problem, consider carefully
  10 ALERT level C = Check. Ensure it is not caused by an omission or oversight
  18 ALERT level G = General information/check it is not something unexpected

   3 ALERT type 1 CIF construction/syntax error, inconsistent or missing data
  10 ALERT type 2 Indicator that the structure model may be wrong or deficient
   6 ALERT type 3 Indicator that the structure quality may be low
   9 ALERT type 4 Improvement, methodology, query or suggestion
   0 ALERT type 5 Informative message, check
```

Datablock: complex4

```
Bond precision:  C-C = 0.0051 A          Wavelength=0.71073

Cell:           a=8.3845(5)       b=8.8813(5)      c=14.9005(11)
                alpha=91.261(5)   beta=93.497(5)   gamma=110.570(5)
Temperature:    293 K
```

	Calculated	Reported
Volume	1035.77(12)	1035.77(12)
Space group	P -1	P -1
Hall group	-P 1	-P 1
Moiety formula	C36 H28 Ag Cl8 N4 O2, F6 Sb	C36 H28 Ag1 Cl8 N4 O2, F6 Sb1
Sum formula	C36 H28 Ag Cl8 F6 N4 O2 Sb	C36 H28 Ag Cl8 F6 N4 O2 Sb
Mr	1175.85	1175.84
Dx,g cm-3	1.885	1.885
Z	1	1
Mu (mm-1)	1.708	1.708
F000	576.0	576.0
F000'	575.93	
h,k,lmax	11,12,20	11,12,20
Nref	6047	6013
Tmin,Tmax	0.921,0.950	0.774,1.000
Tmin'	0.675	

Correction method= # Reported T Limits: Tmin=0.774 Tmax=1.000
AbsCorr = MULTI-SCAN

Data completeness= 0.994 Theta(max)= 29.998

R(reflections)= 0.0404(4135) wR2(reflections)= 0.1005(6013)

S = 1.007 Npar= 265

The following ALERTS were generated. Each ALERT has the format
test-name_ALERT_alert-type_alert-level.
Click on the hyperlinks for more details of the test.

Alert level C

```
PLAT244_ALERT_4_C Low    'Solvent' Ueq as Compared to Neighbors of        Sb1 Check
PLAT334_ALERT_2_C Small Aver. Benzene C-C Dist C6       -C11            1.37 Ang.
PLAT334_ALERT_2_C Small Aver. Benzene C-C Dist C13      -C18            1.37 Ang.
PLAT480_ALERT_4_C Long H...A H-Bond Reported H4       ..F3       .      2.61 Ang.
PLAT910_ALERT_3_C Missing # of FCF Reflection(s) Below Theta(Min).        9 Note
```

Alert level G

```
PLAT154_ALERT_1_G The s.u.'s on the Cell Angles are Equal ..(Note)      0.005 Degree
PLAT199_ALERT_1_G Reported _cell_measurement_temperature ..... (K)        293 Check
PLAT200_ALERT_1_G Reported   _diffrn_ambient_temperature ..... (K)        293 Check
PLAT793_ALERT_4_G Model has Chirality at C1         (Centro SPGR)          R Verify
PLAT794_ALERT_5_G Tentative Bond Valency for Sb1       (V)       .      5.16 Info
PLAT912_ALERT_4_G Missing # of FCF Reflections Above STh/L=  0.600       25 Note
PLAT941_ALERT_3_G Average HKL Measurement Multiplicity ..........       1.9 Low
PLAT978_ALERT_2_G Number C-C Bonds with Positive Residual Density.        1 Info
```

---

0 **ALERT level A** = Most likely a serious problem - resolve or explain
0 **ALERT level B** = A potentially serious problem, consider carefully

```
 5 ALERT level C = Check. Ensure it is not caused by an omission or oversight
 8 ALERT level G = General information/check it is not something unexpected

 3 ALERT type 1 CIF construction/syntax error, inconsistent or missing data
 3 ALERT type 2 Indicator that the structure model may be wrong or deficient
 2 ALERT type 3 Indicator that the structure quality may be low
 4 ALERT type 4 Improvement, methodology, query or suggestion
 1 ALERT type 5 Informative message, check
```

It is advisable to attempt to resolve as many as possible of the alerts in all categories. Often the minor alerts point to easily fixed oversights, errors and omissions in your CIF or refinement strategy, so attention to these fine details can be worthwhile. In order to resolve some of the more serious problems it may be necessary to carry out additional measurements or structure refinements. However, the purpose of your study may justify the reported deviations and the more serious of these should normally be commented upon in the discussion or experimental section of a paper or in the "special_details" fields of the CIF. checkCIF was carefully designed to identify outliers and unusual parameters, but every test has its limitations and alerts that are not important in a particular case may appear. Conversely, the absence of alerts does not guarantee there are no aspects of the results needing attention. It is up to the individual to critically assess their own results and, if necessary, seek expert advice.

Publication of your CIF in IUCr journals

A basic structural check has been run on your CIF. These basic checks will be run on all CIFs submitted for publication in IUCr journals (*Acta Crystallographica*, *Journal of Applied Crystallography*, *Journal of Synchrotron Radiation*); however, if you intend to submit to *Acta Crystallographica Section C* or *E* or *IUCrData*, you should make sure that full publication checks are run on the final version of your CIF prior to submission.

Publication of your CIF in other journals

Please refer to the *Notes for Authors* of the relevant journal for any special instructions relating to CIF submission.

PLATON version of 05/12/2020; check.def file version of 05/12/2020

Datablock complex3 - ellipsoid plot

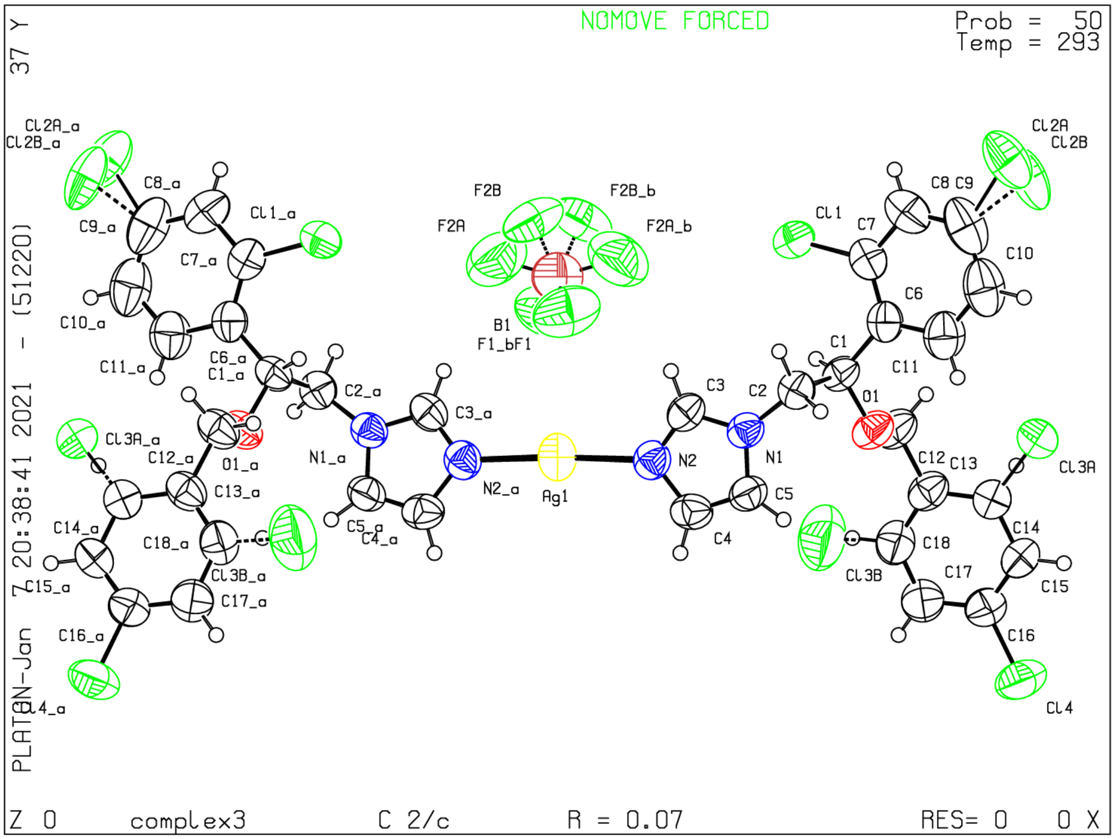

Datablock complex4 - ellipsoid plot

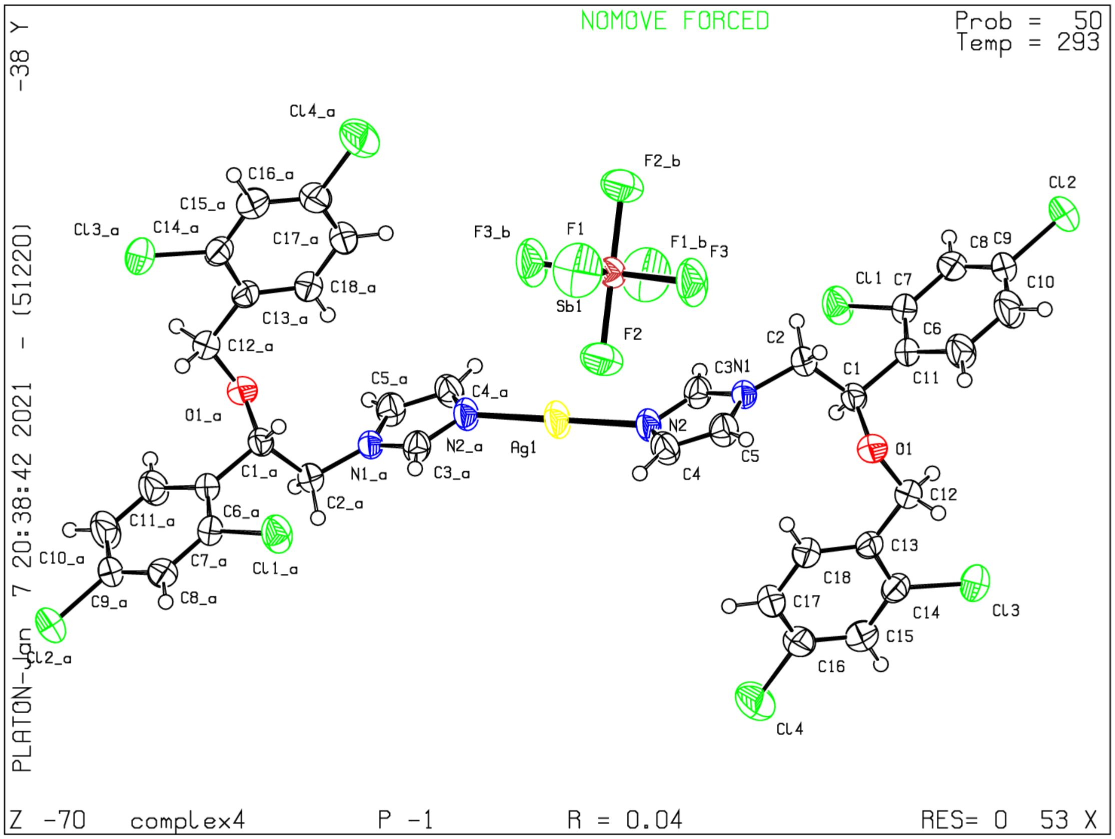

Supplement: Supplementary file 1 [file ijms-22-01510-s001.zip › supplementary files/checkcif-Ag-miconazole-3and4-7Jan2021.pdf]
