# Supplementary material for: Synthesis, Spectroscopy, Single-Crystal Structure Analysis and Antibacterial Activity of Two Novel Complexes of Silver(I) with Miconazole Drug
Source: Int J Mol Sci. 2021 Feb 3;22(4):1510. doi: 10.3390/ijms22041510 (PMC7919260; doi:10.3390/ijms22041510)
Supplement: Supplementary file 1 [file ijms-22-01510-s001.zip › supplementary files/Supplementary Materials.pdf]

Synthesis, spectroscopy, single - crystal structure analysis and antibacterial activity of two novel complexes of silver(I) with miconazole drug

Karolina Stryjska, Izabela Korona-Glowniak, Lilianna Chęcińska, Joachim Kusz and Justyn Ochocki

Contents:

- **Figure S1.** ^{1}H NMR spectra (600 MHz, $CDCl_3$) of miconazole (**a**), $[Ag(MCZ)_2BF_4]$ (**b**), $[Ag(MCZ)_2SbF_6]$ (**c**).
- **Figure S2.** ^{13}C NMR spectra (600 MHz, $CDCl_3$) of miconazole (**a**), $[Ag(MCZ)_2BF_4]$ (**b**), $[Ag(MCZ)_2SbF_6]$ (**c**).
- **Figure S3.** IR spectra of miconazole (**a**), $[Ag(MCZ)_2BF_4]$ (**b**), $[Ag(MCZ)_2SbF_6]$ (**c**).
- **Figure S4.** Different substrates (tissue paper, paper, glass) impregnated with 0.05 mol/L solutions of $AgBF_4$, $[Ag(MCZ)_2BF_4]$, $AgSbF_6$, $[Ag(MCZ)_2SbF_6]$, $AgNO_3$, $[Ag(MCZ)_2NO_3]$, $AgClO_4$, $[Ag(MCZ)_2ClO_4]$, exposed to in normal light at room temperature.

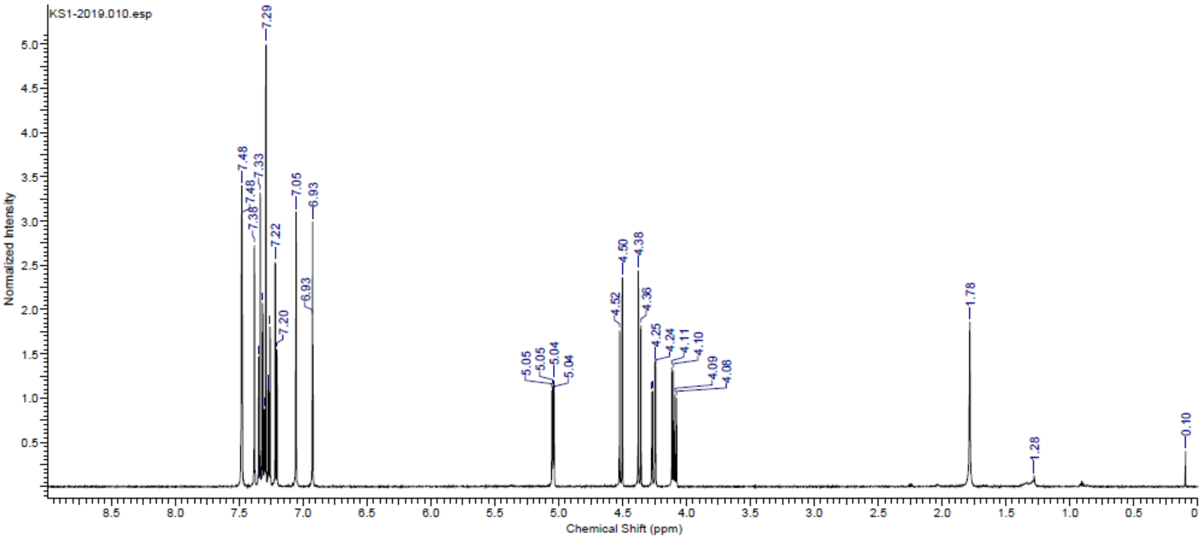

No.	(ppm)	(Hz)	Height
1	0.10	58.1	0.4007
2	1.28	768.5	0.1182
3	1.78	1071.1	1.8607
4	4.08	2447.8	1.0000
5	4.09	2455.3	1.0349
6	4.10	2462.5	1.3126
7	4.11	2470.0	1.3460
8	4.24	2546.1	1.3542
9	4.25	2548.7	1.4103
10	4.27	2560.7	1.0808
11	4.27	2563.4	1.0673
12	4.36	2615.0	1.8211
13	4.38	2627.8	2.4370
14	4.50	2702.7	2.3633
15	4.52	2715.5	1.7647
16	5.04	3022.7	1.0541
17	5.04	3025.3	1.1984
18	5.05	3030.2	1.1333
19	5.05	3032.8	1.0872
20	6.93	4157.3	2.9896
21	6.93	4158.4	1.9196
22	7.05	4234.8	3.1061
23	7.20	4324.4	1.5479
24	7.22	4332.7	2.5349
25	7.26	4355.7	1.7937
26	7.26	4357.5	1.8076
27	7.27	4363.9	1.0690
28	7.27	4365.8	1.1503
29	7.29	4376.4	5.0000
30	7.30	4381.6	0.8576
31	7.30	4383.5	0.8793
32	7.31	4389.9	1.9547
33	7.32	4391.8	2.0716
34	7.33	4397.1	0.1077
35	7.33	4402.7	3.3216
36	7.35	4411.0	1.4680
37	7.38	4429.1	2.7270
38	7.38	4431.3	2.6959
39	7.48	4487.4	3.0263
40	7.48	4489.7	3.3984
41	7.48	4491.2	3.0817

(a)

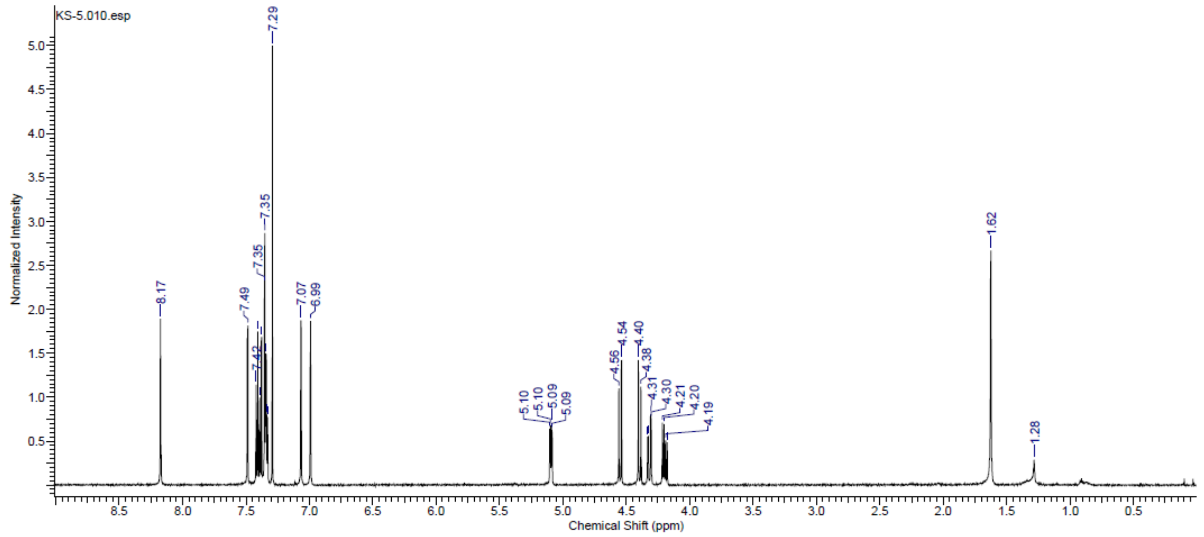

No.	(ppm)	(Hz)	Height	No.	(ppm)	(Hz)	Height	No.	(ppm)	(Hz)	Height	No.	(ppm)	(Hz)	Height	No.	(ppm)	(Hz)	Height
1	1.28	770.0	0.2880	8	4.31	2585.2	0.8045	15	5.09	3052.4	0.6235	22	7.33	4397.8	0.5744	29	7.38	4428.3	1.6843
2	1.62	975.1	2.6670	9	4.33	2597.3	0.5575	16	5.09	3055.0	0.7067	23	7.33	4400.1	0.7710	30	7.39	4436.6	0.9929
3	4.18	2507.3	0.4849	10	4.33	2599.9	0.5483	17	5.10	3060.3	0.6758	24	7.34	4403.8	0.8002	31	7.41	4445.3	1.7454
4	4.19	2515.2	0.5079	11	4.38	2631.1	1.1204	18	5.10	3062.9	0.6372	25	7.34	4406.1	1.4948	32	7.42	4453.5	1.1413
5	4.20	2522.0	0.6918	12	4.40	2643.9	1.4205	19	6.99	4196.4	1.8628	26	7.34	4408.0	1.4747	33	7.49	4493.4	1.8150
6	4.21	2529.9	0.7086	13	4.54	2723.8	1.4217	20	7.07	4241.2	1.8718	27	7.35	4412.5	2.8644	34	7.49	4495.3	1.6696
7	4.30	2582.6	0.7541	14	4.56	2736.2	1.0974	21	7.29	4376.7	5.0000	28	7.35	4414.4	2.2944	35	8.17	4906.4	1.8892

(b)

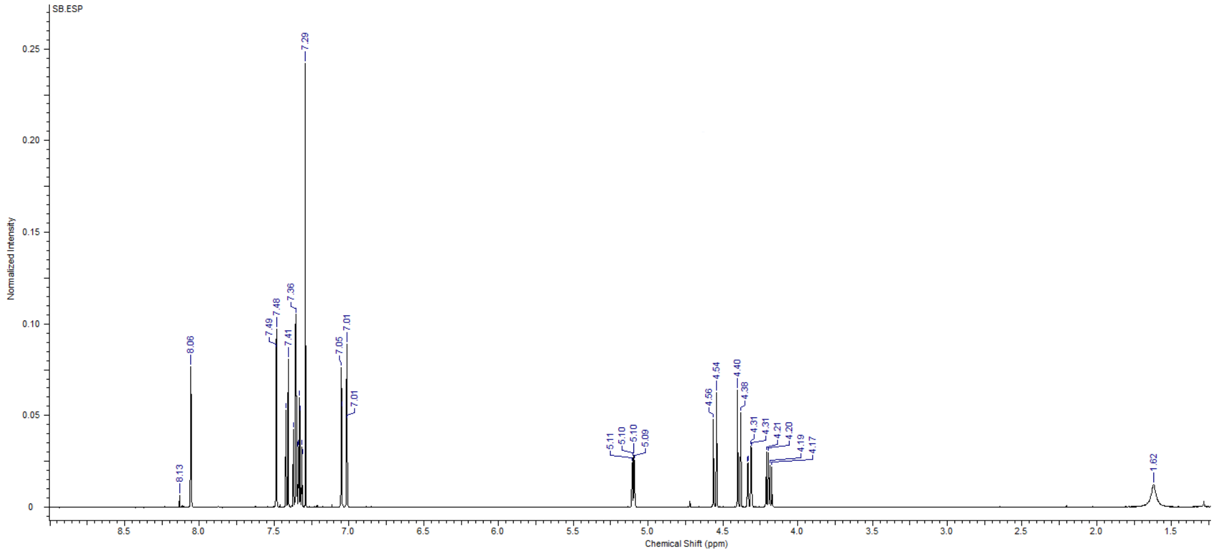

No.	(ppm)	(Hz)	Height	No.	(ppm)	(Hz)	Height	No.	(ppm)	(Hz)	Height	No.	(ppm)	(Hz)	Height	No.	(ppm)	(Hz)	Height
1	-0.07	-42.8	0.0173	11	4.21	2526.9	0.0301	21	5.10	3058.4	0.0281	31	7.31	4389.2	0.0277	41	7.41	4446.0	0.0808
2	0.02	14.1	0.1564	12	4.31	2585.6	0.0323	22	5.10	3063.3	0.0270	32	7.32	4391.4	0.0328	42	7.42	4454.7	0.0529
3	0.03	20.8	0.1301	13	4.31	2588.2	0.0333	23	5.11	3066.3	0.0244	33	7.33	4397.4	0.0515	43	7.48	4492.7	0.0970
4	0.04	23.8	0.0062	14	4.33	2600.3	0.0242	24	7.01	4208.8	0.0474	34	7.33	4399.7	0.0599	44	7.49	4494.6	0.0860
5	0.10	60.0	0.0110	15	4.34	2602.9	0.0236	25	7.01	4210.4	0.0887	35	7.34	4404.2	0.0327	45	8.06	4835.6	0.0765
6	0.13	75.8	0.0168	16	4.38	2629.6	0.0513	26	7.02	4211.5	0.0512	36	7.34	4406.1	0.0321	46	8.13	4882.3	0.0062
7	1.62	972.1	0.0126	17	4.40	2642.1	0.0640	27	7.05	4230.7	0.0537	37	7.35	4412.5	0.0527				
8	4.17	2504.7	0.0217	18	4.54	2726.4	0.0626	28	7.05	4231.8	0.0763	38	7.35	4413.6	0.1048				
9	4.19	2512.6	0.0227	19	4.56	2738.8	0.0479	29	7.05	4233.3	0.0482	39	7.36	4415.9	0.1055				
10	4.20	2519.0	0.0295	20	5.09	3055.4	0.0256	30	7.29	4376.7	0.2422	40	7.37	4424.9	0.0423				

(c)

Figure S1. ^{1}H NMR spectra (600 MHz, $CDCl_3$) of miconazole (**a**), $[Ag(MCZ)_2BF_4]$ (**b**), $[Ag(MCZ)_2SbF_6]$ (**c**).

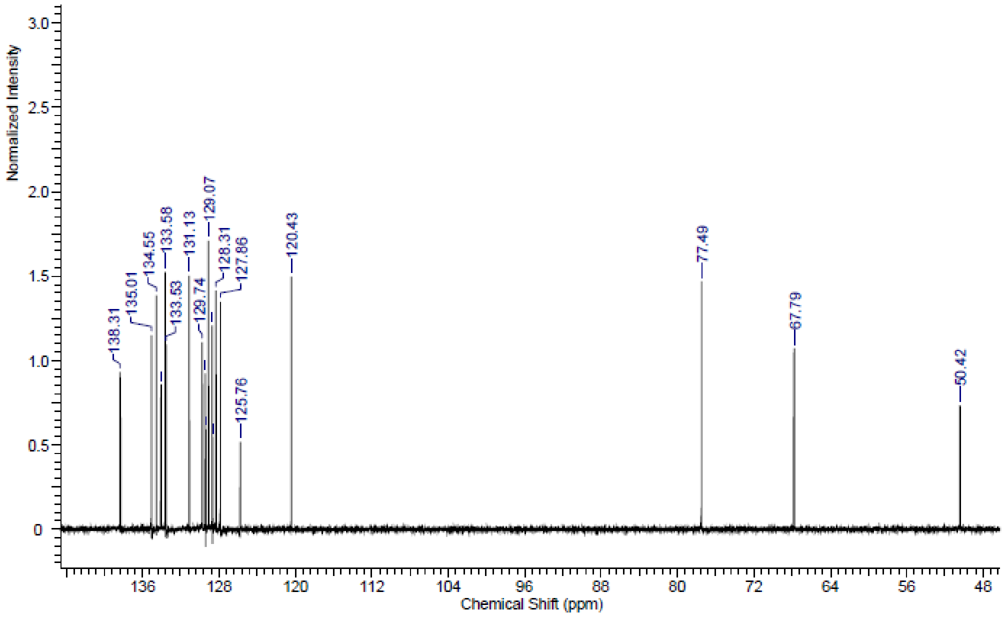

No.	(ppm)	(Hz)	Height	No.	(ppm)	(Hz)	Height	No.	(ppm)	(Hz)	Height
1	0.55	82.6	0.6551	11	67.79	10232.6	1.0718	21	129.44	19538.6	0.9225
2	21.52	3248.4	0.1346	12	77.49	11697.2	1.4683	22	129.74	19584.9	1.1059
3	39.59	5976.3	0.6640	13	120.43	18178.6	1.4941	23	131.13	19793.9	1.5042
4	39.73	5997.2	2.1278	14	125.76	18984.0	0.5161	24	133.53	20156.0	1.0935
5	39.87	6018.1	4.2669	15	127.86	19299.9	1.3490	25	133.58	20163.7	1.5257
6	40.01	6039.0	5.0000	16	128.31	19368.1	1.4133	26	134.09	20240.7	0.8566
7	40.15	6059.9	4.2066	17	128.65	19419.8	0.5427	27	134.55	20310.0	1.3828
8	40.28	6080.8	2.0824	18	128.70	19427.5	1.2053	28	135.01	20380.4	1.1499
9	40.42	6101.7	0.6717	19	129.07	19483.6	1.7088	29	138.31	20877.8	0.9302
10	50.42	7610.4	0.7365	20	129.35	19525.4	0.5928				

(a)

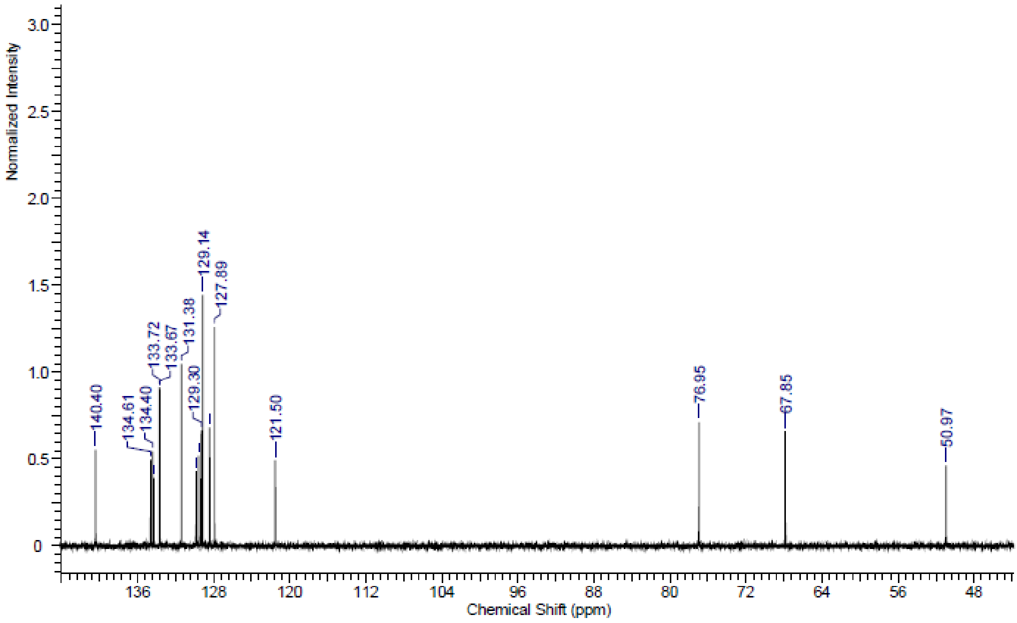

No.	(ppm)	(Hz)	Height	No.	(ppm)	(Hz)	Height	No.	(ppm)	(Hz)	Height
1	0.56	84.8	0.2123	10	67.85	10242.5	0.6582	19	131.38	19832.4	1.0464
2	39.60	5977.4	0.6453	11	76.95	11615.8	0.7116	20	133.67	20178.0	0.9023
3	39.74	5998.3	2.0681	12	121.50	18340.3	0.4902	21	133.72	20184.6	0.9121
4	39.88	6019.2	4.2216	13	127.89	19305.4	1.2610	22	134.31	20274.8	0.3903
5	40.01	6040.1	5.0000	14	128.42	19385.7	0.6838	23	134.40	20288.0	0.5445
6	40.15	6061.0	4.2534	15	129.14	19493.5	1.4434	24	134.61	20319.9	0.4966
7	40.29	6081.9	2.1408	16	129.30	19517.7	0.6641	25	140.40	21193.6	0.5520
8	40.43	6102.8	0.6962	17	129.56	19557.3	0.5185				
9	50.97	7694.0	0.4621	18	129.82	19597.0	0.4277				

(b)

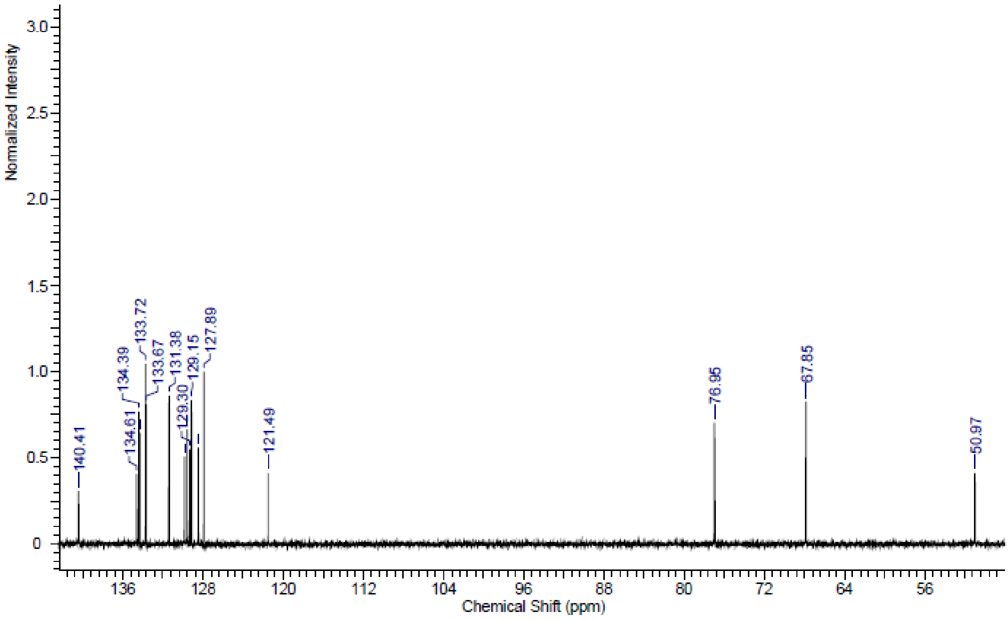

No.	(ppm)	(Hz)	Height	No.	(ppm)	(Hz)	Height	No.	(ppm)	(Hz)	Height
1	0.56	84.8	0.4424	10	67.85	10242.5	0.8254	19	131.38	19832.4	0.8616
2	39.59	5976.3	0.7107	11	76.95	11615.8	0.7007	20	133.67	20178.0	0.8111
3	39.73	5997.2	2.1503	12	121.49	18339.2	0.4127	21	133.72	20184.6	1.0447
4	39.87	6018.1	4.1851	13	127.89	19305.4	0.9960	22	134.31	20274.8	0.6438
5	40.01	6040.1	5.0000	14	128.42	19385.7	0.5580	23	134.39	20286.9	0.7678
6	40.15	6061.0	4.3612	15	129.15	19494.6	0.8329	24	134.61	20319.9	0.4044
7	40.29	6081.9	2.2479	16	129.30	19517.7	0.5472	25	140.41	21194.7	0.3084
8	40.43	6102.8	0.7457	17	129.57	19558.4	0.6627				
9	50.97	7694.0	0.4112	18	129.81	19594.8	0.5059				

(c)

Figure S2. ^{13}C NMR spectra (600 MHz, $CDCl_3$) of miconazole (**a**), $[Ag(MCZ)_2BF_4]$ (**b**), $[Ag(MCZ)_2SbF_6]$ (**c**).

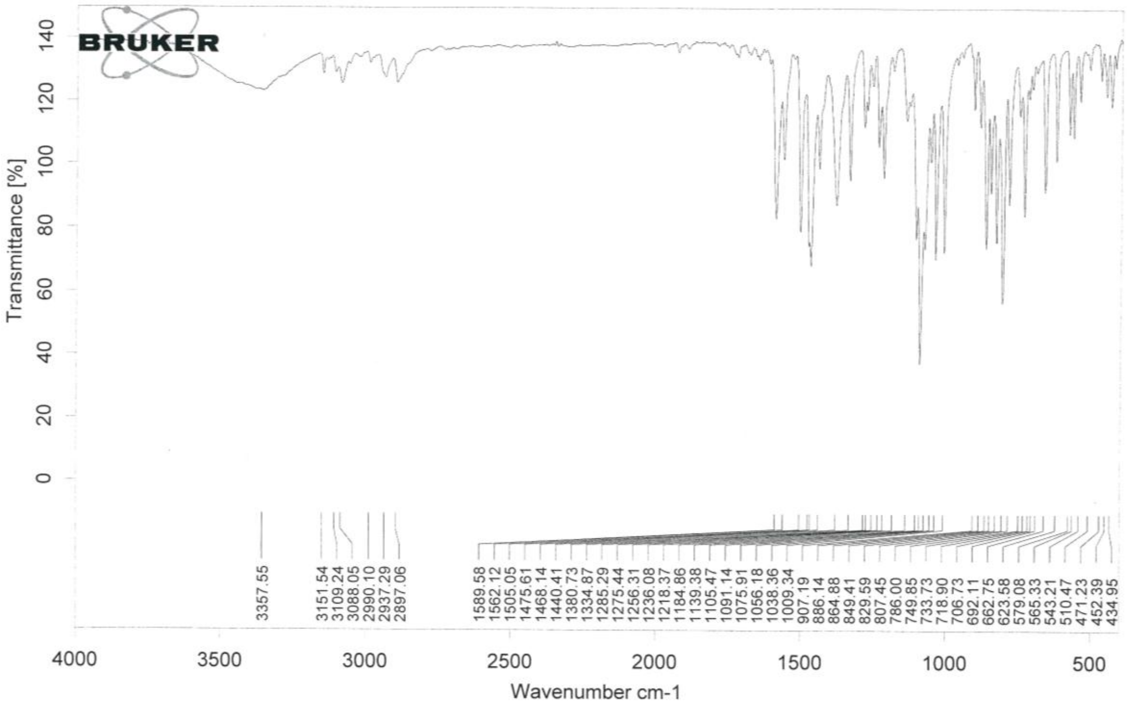

(a)

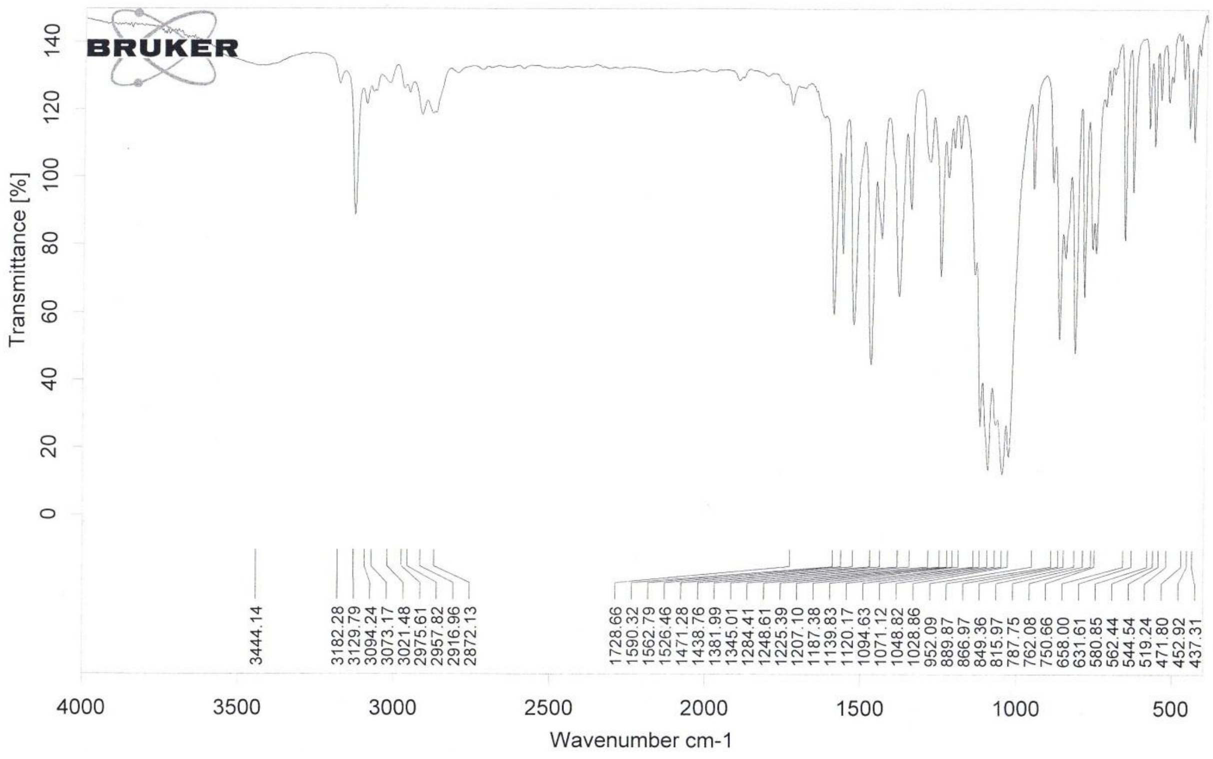

(b)

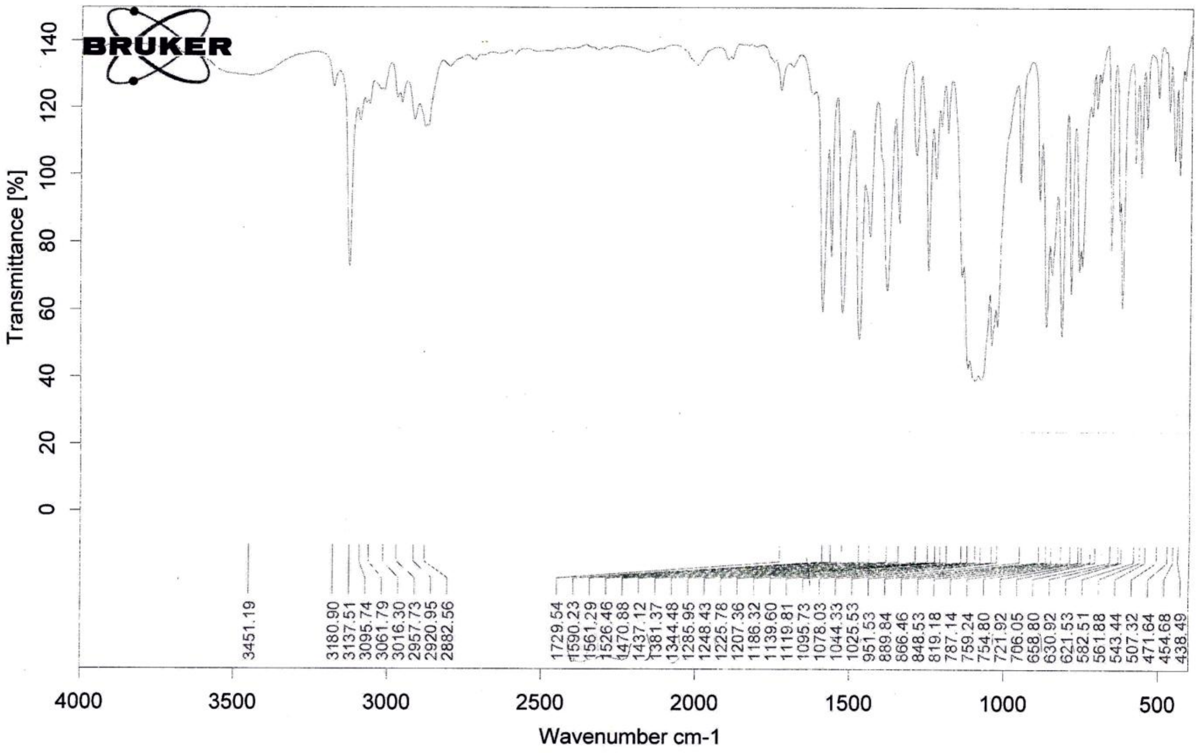

(c)

Figure S3. IR spectra of miconazole (**a**), [Ag(MCZ)$_2$BF$_4$] (**b**), [Ag(MCZ)$_2$SbF$_6$] (**c**).

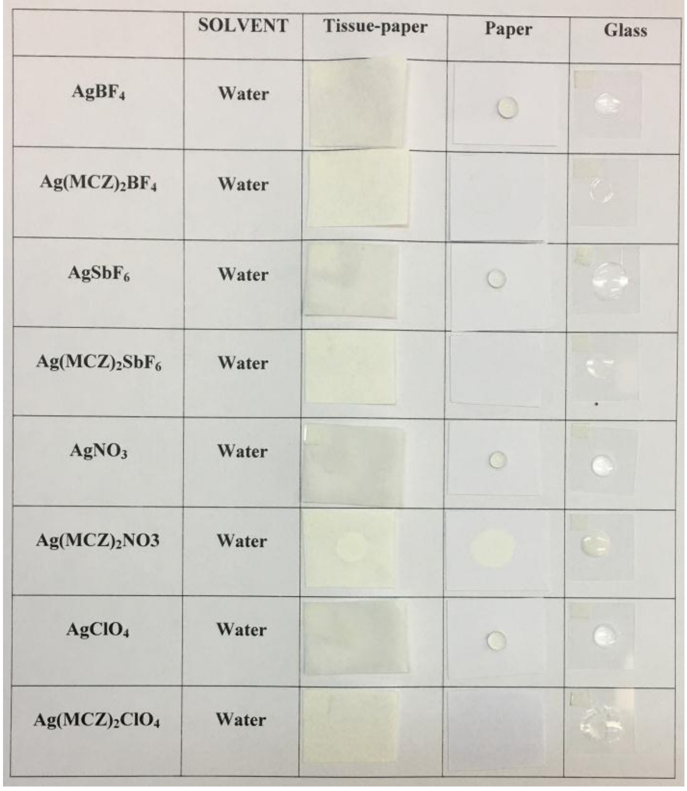

exposition time **0h**

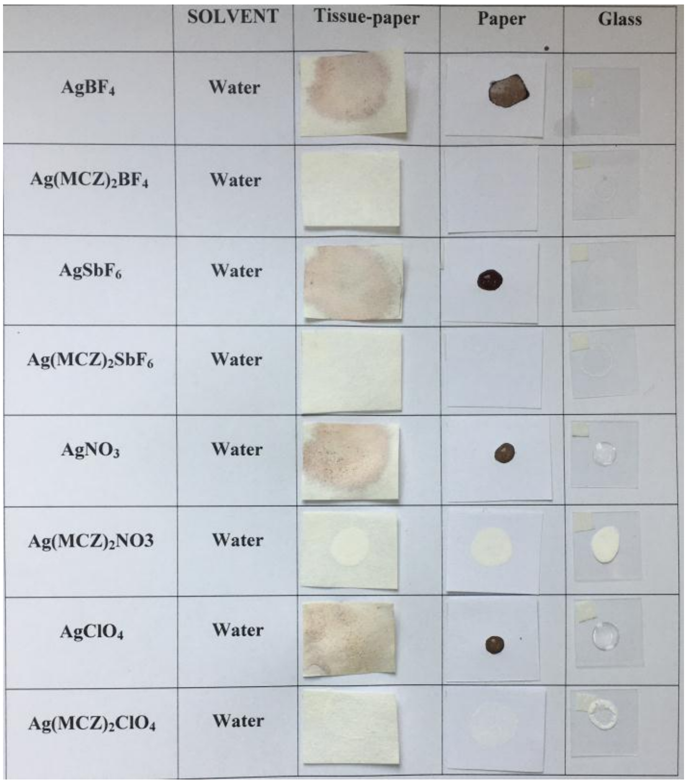

exposition time **1h**

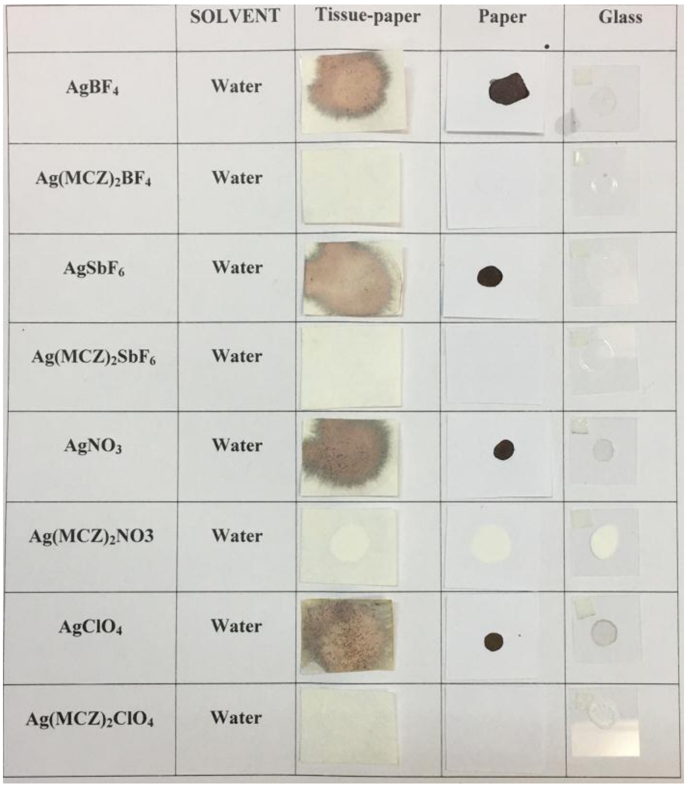

exposition time **4h**

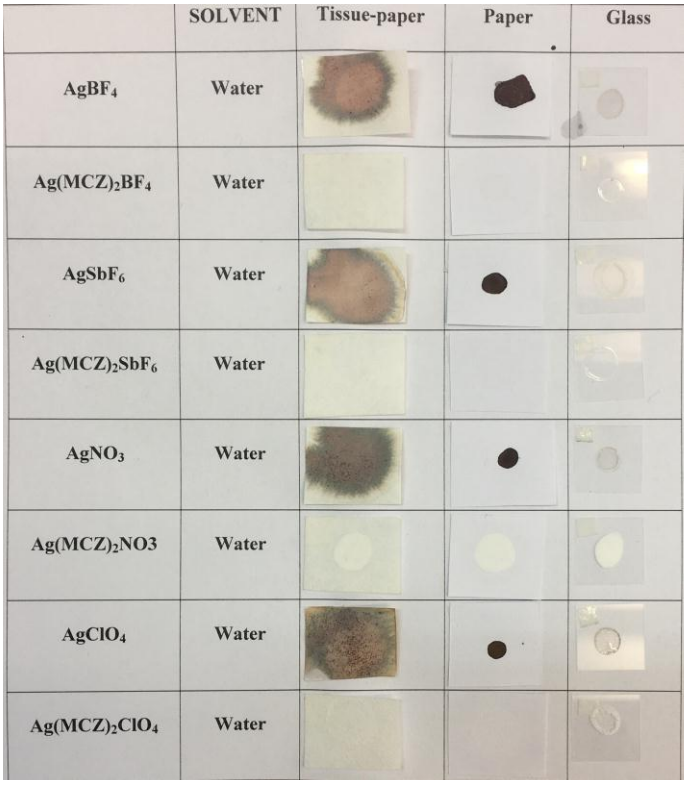

exposition time **18h**

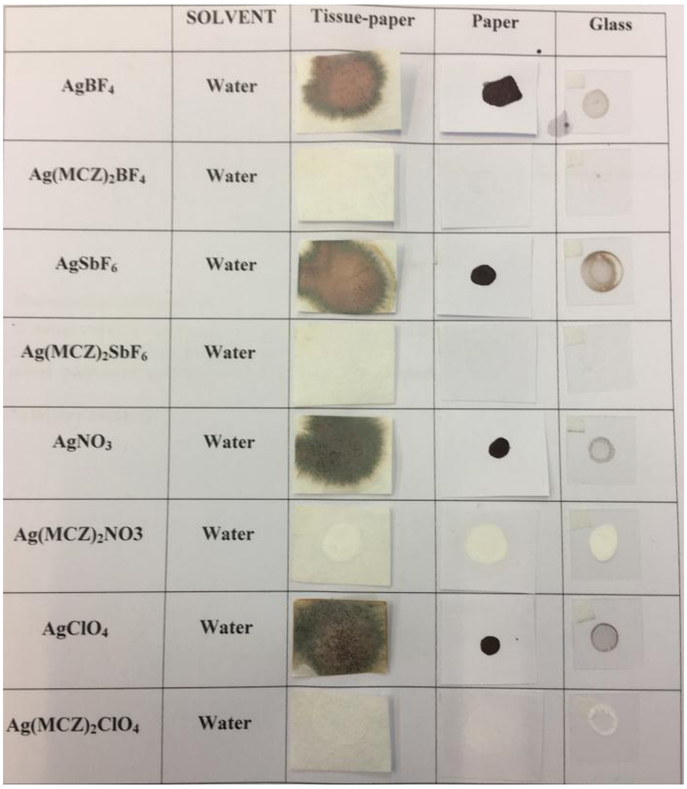

exposition time **24h**

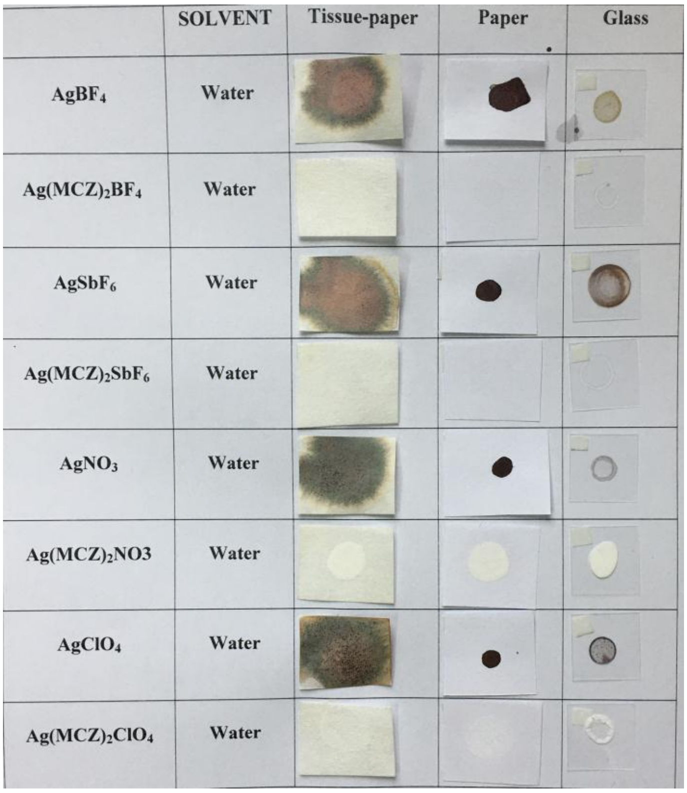

exposition time **40h**

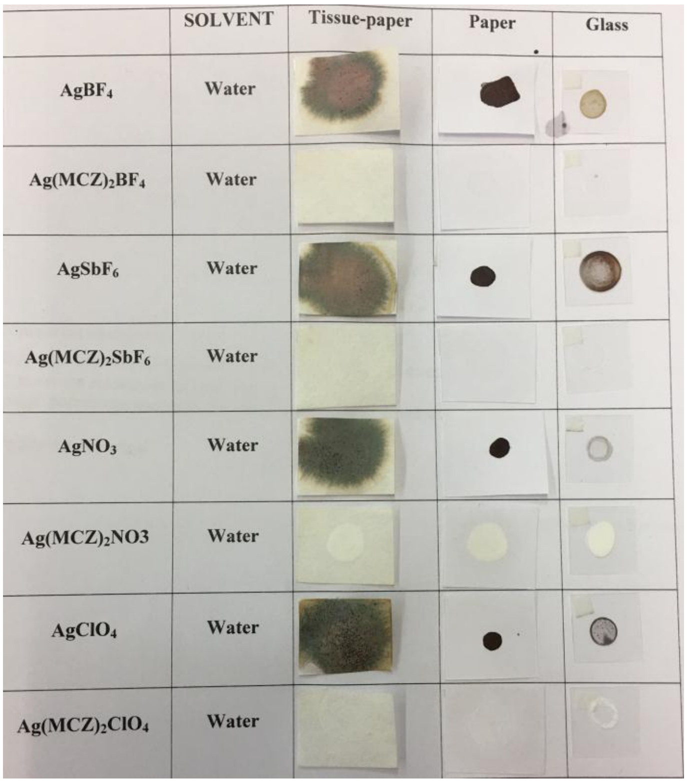

exposition time **48h**

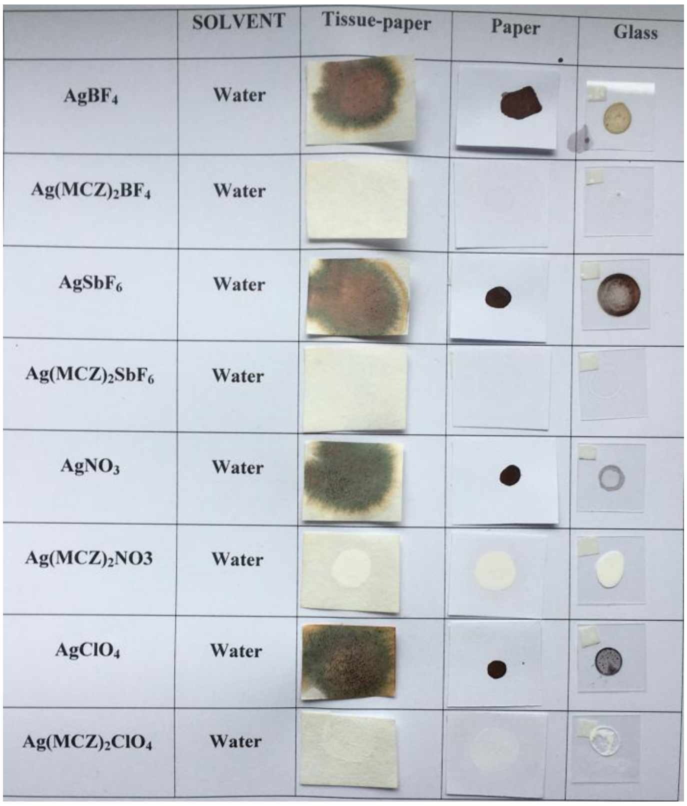

exposition time **52h**

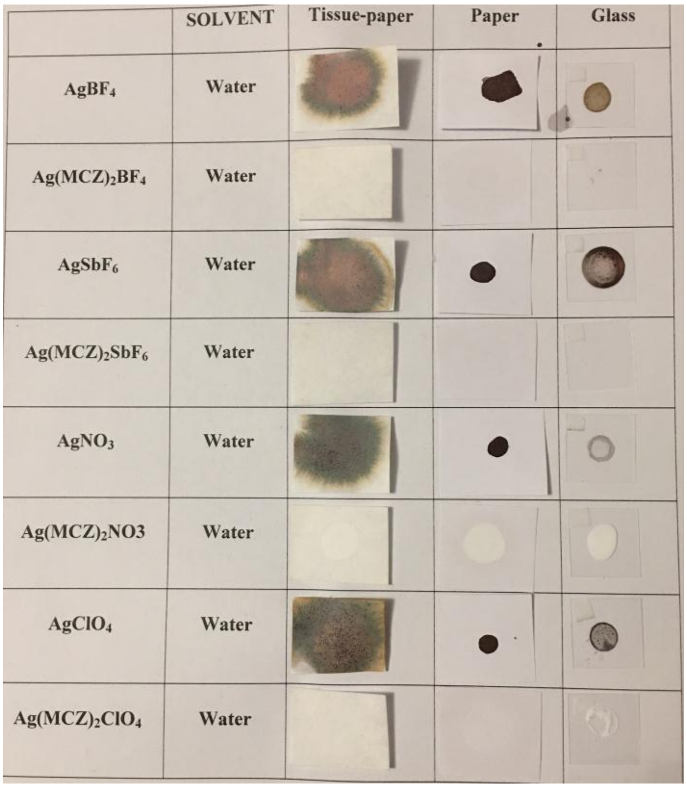

exposition time **60h**

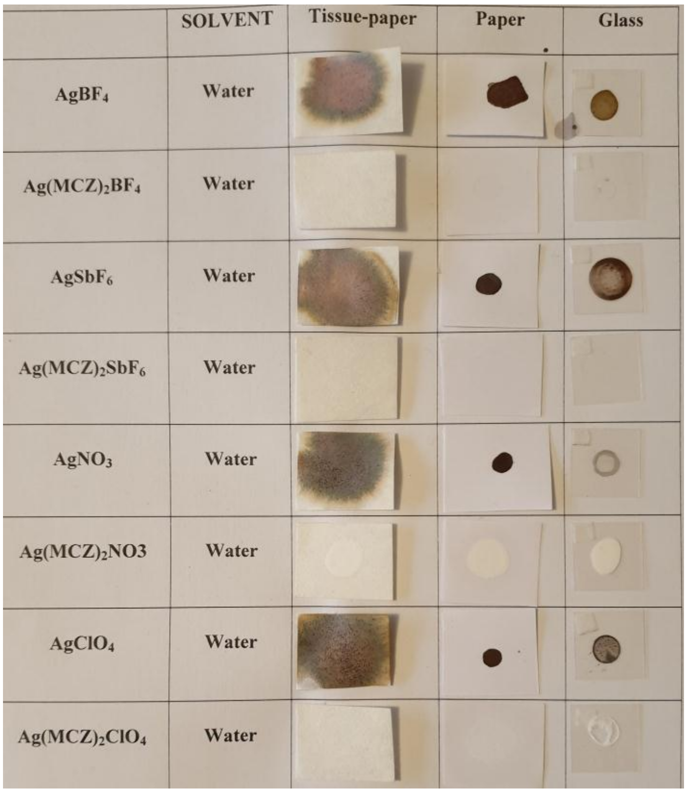

exposition time **84h**

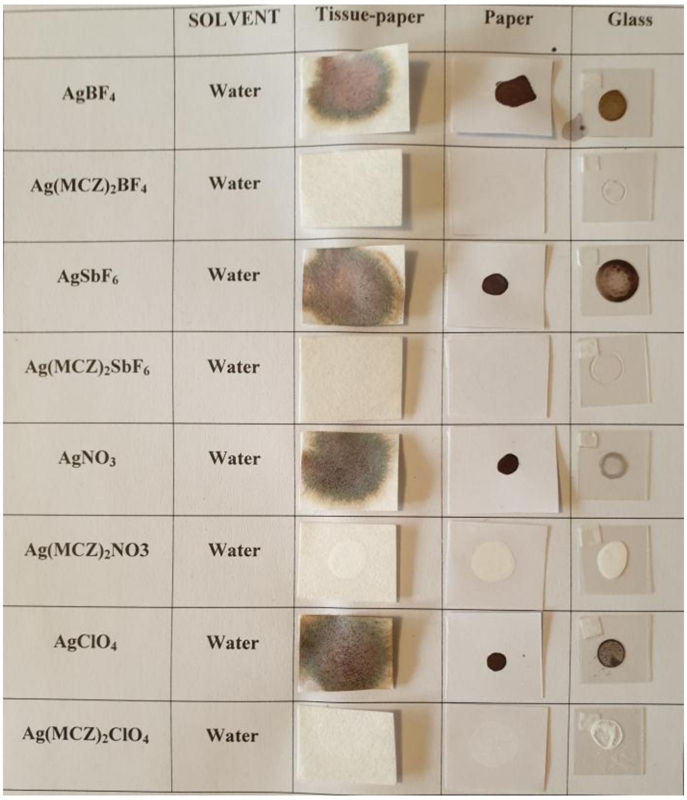

exposition time **108h**

Figure S4. Different substrates (tissue paper, paper, glass) impregnated with 0.05 mol/L solutions of $AgBF_4$, $[Ag(MCZ)_2BF_4]$, $AgSbF_6$, $[Ag(MCZ)_2SbF_6]$, $AgNO_3$, $[Ag(MCZ)_2NO_3]$, $AgClO_4$, $[Ag(MCZ)_2ClO_4]$, exposed to in normal light at room temperature.
