# Supplementary figures and images for: Synthesis, Spectroscopy, Single-Crystal Structure Analysis and Antibacterial Activity of Two Novel Complexes of Silver(I) with Miconazole Drug
Source: Int J Mol Sci. 2021 Feb 3;22(4):1510. doi: 10.3390/ijms22041510 (PMC7919260; doi:10.3390/ijms22041510)

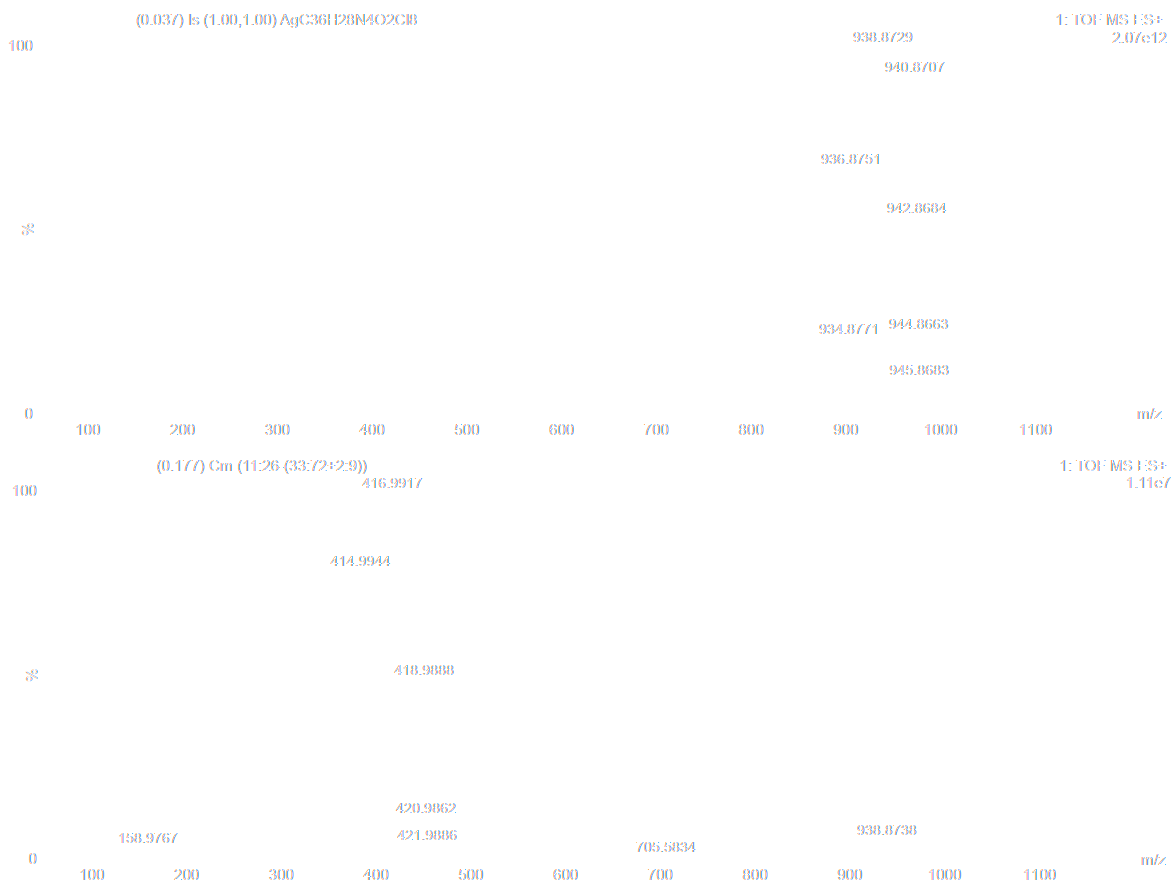

Supplement: Supplementary file 1 [file ijms-22-01510-s001.zip › supplementary files/Ag(MCZ)2BF4 - ESI-mass spectra.PNG]

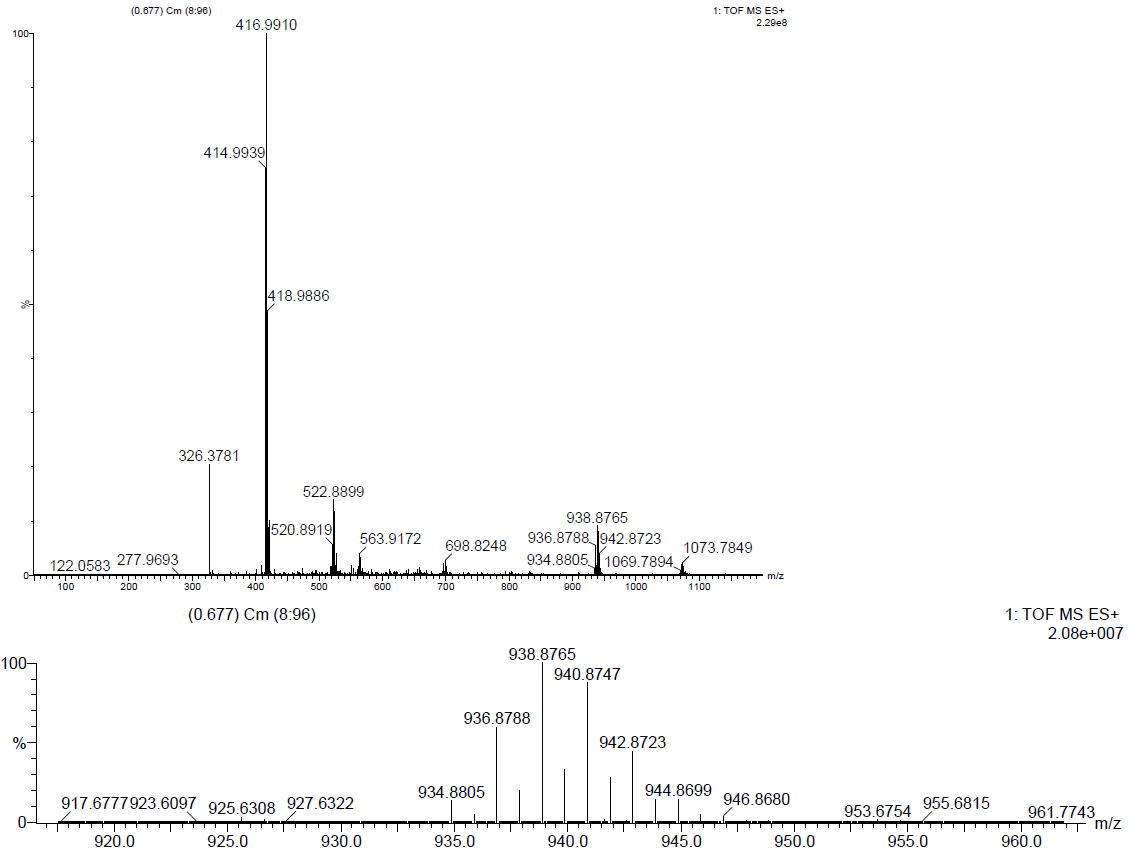

Supplement: Supplementary file 1 [file ijms-22-01510-s001.zip › supplementary files/Ag(MCZ)2SbF6 - ESI-mass spectra.PNG]
